# Identification of coherent patterns in gene expression data using an efficient biclustering algorithm and parallel coordinate visualization

**DOI:** 10.1186/1471-2105-9-210

**Published:** 2008-04-23

**Authors:** Kin-On Cheng, Ngai-Fong Law, Wan-Chi Siu, Alan Wee-Chung Liew

**Affiliations:** 1 Centre for Signal Processing, Department of Electronic and Information Engineering, The Hong Kong Polytechnic University, Hung Hom, Hong Kong, China; 2 School of Information and Communication Technology, Griffith University, Gold Coast Campus, QLD 4222, Queensland, Australia

## Abstract

**Background:**

The DNA microarray technology allows the measurement of expression levels of thousands of genes under tens/hundreds of different conditions. In microarray data, genes with similar functions usually co-express under certain conditions only [1]. Thus, biclustering which clusters genes and conditions simultaneously is preferred over the traditional clustering technique in discovering these coherent genes. Various biclustering algorithms have been developed using different bicluster formulations. Unfortunately, many useful formulations result in NP-complete problems. In this article, we investigate an efficient method for identifying a popular type of biclusters called additive model. Furthermore, parallel coordinate (PC) plots are used for bicluster visualization and analysis.

**Results:**

We develop a novel and efficient biclustering algorithm which can be regarded as a greedy version of an existing algorithm known as pCluster algorithm. By relaxing the constraint in homogeneity, the proposed algorithm has polynomial-time complexity in the worst case instead of exponential-time complexity as in the pCluster algorithm. Experiments on artificial datasets verify that our algorithm can identify both additive-related and multiplicative-related biclusters in the presence of overlap and noise. Biologically significant biclusters have been validated on the yeast cell-cycle expression dataset using Gene Ontology annotations. Comparative study shows that the proposed approach outperforms several existing biclustering algorithms. We also provide an interactive exploratory tool based on PC plot visualization for determining the parameters of our biclustering algorithm.

**Conclusion:**

We have proposed a novel biclustering algorithm which works with PC plots for an interactive exploratory analysis of gene expression data. Experiments show that the biclustering algorithm is efficient and is capable of detecting co-regulated genes. The interactive analysis enables an optimum parameter determination in the biclustering algorithm so as to achieve the best result. In future, we will modify the proposed algorithm for other bicluster models such as the coherent evolution model.

## Background

### Gene expression matrix

**Figure 1:**
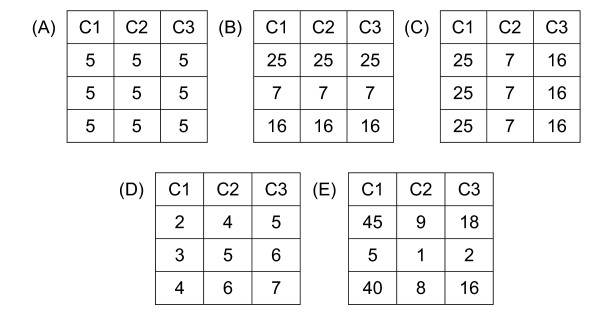
**Examples of different biclusters**. (A) A constant bicluster. (B) A constant row bicluster. (C) A constant column bicluster. (D) An additive-related bicluster. (E) A multiplicative-related bicluster. Note that *Ci *denotes the *i-*th experimental condition.

Data from microarray experiments [2,3] is frequently given as a large matrix showing expression levels of genes (rows) under different experimental conditions (columns). The so-called gene expression data can thus be written as a matrix of size *m *× *n *where *m *is the number of genes and *n *is the number of experimental conditions. Typically *m *is much greater than *n*. For example, (*m*, *n*) is (6220, 15) and (4026, 47) respectively for the time series yeast samples [4] and lymphoma specimens [5]. One of the challenges in microarray data analysis is to identify groupings of genes with similar behaviours/functions. Several clustering algorithms have been applied to DNA gene expression data to identify biologically relevant groupings based on similarity in expression profiles [6-10]. However, traditional clustering techniques are global in nature in which the expression patterns are grouped either along the entire row or along the entire column [1,11]. This implies that one would find the grouping of genes that would express similarly for all conditions, or the groupings of conditions in which all genes exhibit similar behaviour. However, in practice only a subset of genes is highly correlated under a subset of conditions. This requires simultaneous clustering along both the row and column directions, and is often called biclustering [11-16]. A bicluster often exhibit certain kinds of homogeneity, for example constant level of expression throughout the whole bicluster (constant bicluster), constant level of expression along either rows or columns (constant rows and constant columns), and rows/columns that are related by additions or multiplications [15], as shown in Figure 1. We have recently shown that the different bicluster patterns have a simple geometric interpretation as linear objects in a high dimensional feature space [14,15]. A comprehensive survey on different biclustering algorithms was given in references [11,13,16].

### Parallel coordinate plots

The parallel coordinate (PC) technique is a powerful method for visualizing and analyzing high-dimensional data under a two-dimensional setting [17,18]. In this technique, each dimension is represented as a vertical axis, and then the *N*-dimensional axis is arranged in parallel to each other. By giving up the orthogonal representation, the number of dimensions that can be visualized is not restricted to only two [19-21]. Studies have found that geometric structure can still be preserved by the PC plot despite that the orthogonal property is destroyed [17-21]. In gene expression matrix, each gene is represented by a vector of conditions (i.e., row) and each condition is considered as a vector of genes (i.e., column). Since gene expression data always involves a large number of genes as well as a certain number of experimental conditions, the PC technique is well suited to their analysis. Moreover, visualization of gene expression data is an important problem for biological knowledge discovery [22]. Thus, the PC plots have been studied for gene expression data visualization [23,24]. Further details about visualization of biclusters using PC plots are provided in Additional file 1. In section "Method", a new greedy algorithm for bicluster identification is presented. Meanwhile, an interactive approach of parameter determination for the proposed biclustering algorithm based on PC visualization is discussed.

## Methods

### Identification of biclusters from difference matrix

**Figure 2:**
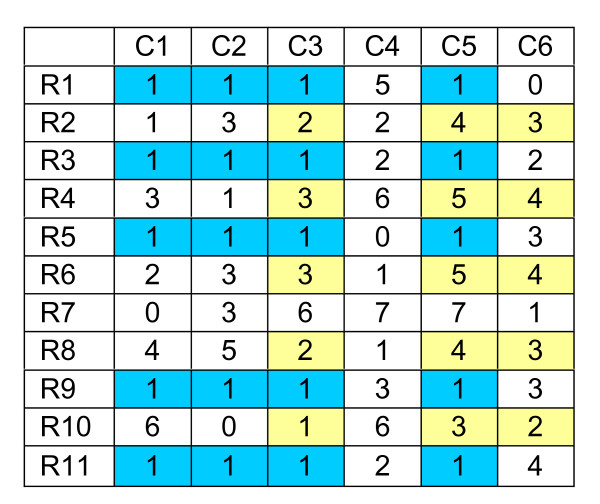
An example of gene expression matrix with two embedded biclusters.

**Figure 3:**
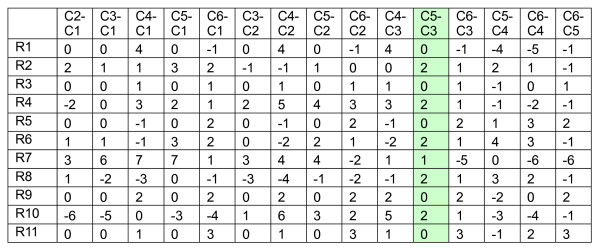
The difference matrix for the dataset shown in Figure 2.

The biclusters given in Figure 1(A)–(D) can be described by an additive model in which each pair of rows has the same difference in all the related columns or each pair of columns has the same difference in all the related rows. Thus, a difference matrix, each column of which represents the column differences between a pair of columns in a data matrix, provides useful information for identification of additive-related biclusters. Consider the data in Figure 2, there are two biclusters: the first one (shown in blue color) is a constant bicluster while the second one (shown in yellow color) is an additive-related bicluster. As the rows in a bicluster is supposed to correlate in a subset of columns, the column difference between every two columns is computed so as to identify this column subset. There are altogether 6(6-1)/2 = 15 permutations as shown in the difference matrix in Figure 3. In the difference matrix, we can find special features that are related to the biclusters. For example, consider column "C5-C3". There are only three distinct difference values: 0 (5 counts), 1 (1 count), 2 (5 counts). This suggests the existence of three biclusters formed between "C5" and "C3":

• the first bicluster is for rows R1, R3, R5, R9 and R11 in which the difference between "C5" and "C3" is zero, i.e., a constant bicluster;

• the second bicluster is for rows R2, R4, R6, R8 and R10 in which the difference between "C5" and "C3" is two, i.e., an additive bicluster; and

• the third bicluster involves row R7 only, thus it is not considered to be a valid bicluster.

**Figure 4:**
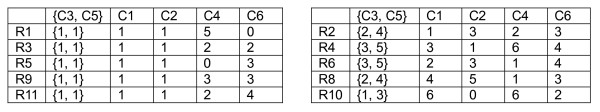
The two different groups formed by merging columns "C5" and "C3".

**Figure 5:**
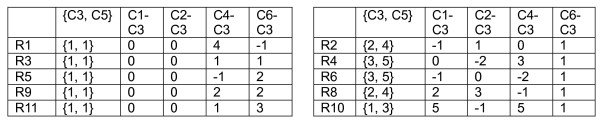
The difference matrix for the two different groups formed by merging columns "C5" and "C3".

Analyzing the distribution along the column direction in the difference matrix thus helps to identify possible biclusters. In the above example, we have two valid biclusters. Thus, C3 and C5 are merged to form two groups as shown in Figure 4. The analysis can be repeated for each of these two groups to find out whether any other columns can be merged to {C3, C5}, i.e., using either C3 or C5 as a reference, we check whether C1, C2, C4 and C6 can be merged with {C3, C5}. In particular, if C3 is used as a reference, two difference matrices as shown in Figure 5 can be obtained. Note that their difference values can be read directly from the original difference matrix of Figure 3. By examining the first difference matrix in Figure 5, we see that two paired columns, "C1-C3" and "C2-C3", show a single bicluster with a difference value equals to zero. This suggests that columns C1 and C2 can be merged to {C3, C5} for rows R1, R3, R5, R9 and R11. The second difference matrix also has a single cluster with a difference value equal to 1 at paired column "C6-C3". Therefore, C6 can be merged to {C3, C5} for rows R2, R4, R6, R8 and R10. Thus by this repeated bicluster growing process – expanding the column set and refining the row set, we can identify possible biclusters embedded in the dataset. Also, note that the difference matrix needs to be calculated only once. This greatly reduces the computational complexity of our algorithm.

### Proposed algorithm for additive models

Additive-related biclusters can be found by progressively merging columns through studying the data distribution along each column in the difference matrix. If there is just one bicluster between two columns in the gene expression matrix, the distribution will have a single peak in one of the columns of the difference matrix. Related rows for this bicluster can then be identified. If there are multiple biclusters formed between two columns in the gene expression matrix, we can separate the rows into different groups by examining the distribution in the corresponding columns of the difference matrix. Therefore, by analyzing the distributions of difference values along columns of the difference matrix, peaks that correspond to different biclusters can be identified.

**Figure 6:**
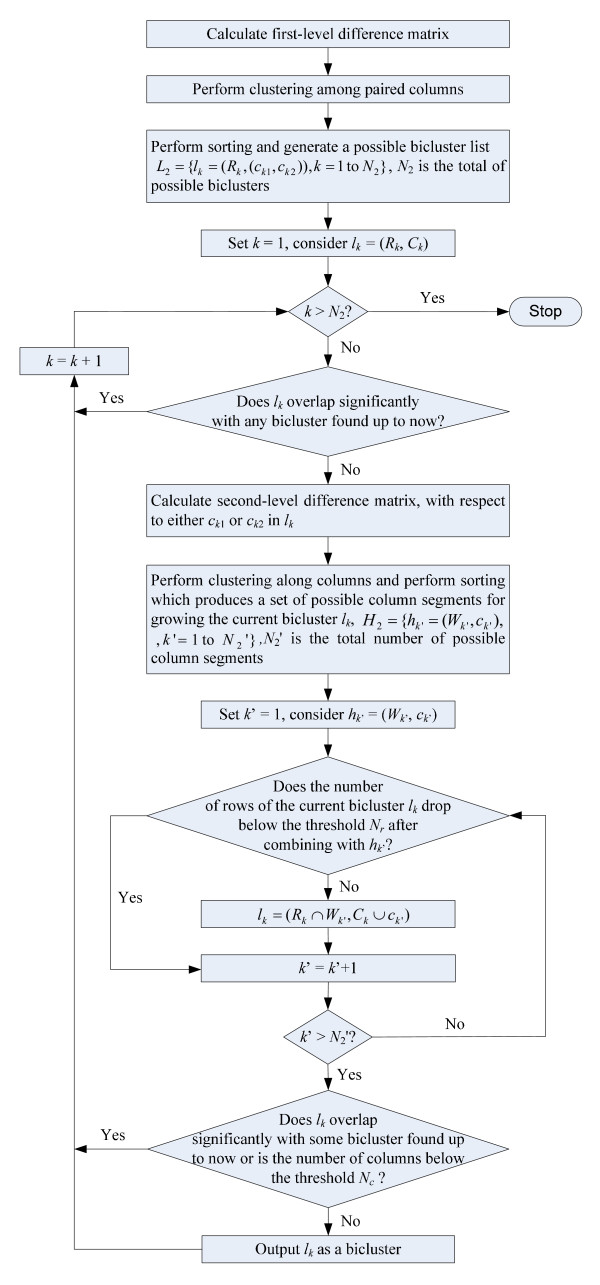
The flow chart of the proposed biclustering algorithm.

**Figure 7:**
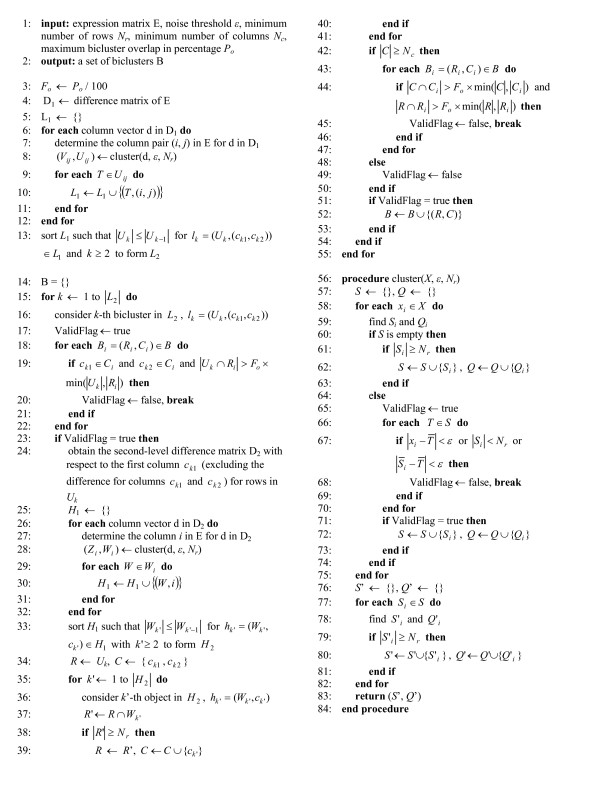
The pseudo-code of the proposed biclustering algorithm.

An overview of the procedure of our proposed biclustering algorithm is shown in the flow chart provided in Figure 6 while the details are described in the pseudo-code in Figure 7. There are four parameters in our algorithm: noise threshold *ε*, minimum number of rows *N*_*r*_, minimum number of columns *N*_*c *_and maximum bicluster overlap in percentage *P*_*o*_. The parameter *ε *specifies the noise tolerance as well as the homogeneity in the identified biclusters. On the other hand, *N*_*r *_and *N*_*c *_set the lower bounds of the number of rows and columns of the identified biclusters respectively. *P*_*o *_determines the maximum degree of overlap between identified biclusters. More specifically, no overlap exceeding *P*_*o *_percentage in both the row and column dimensions simultaneously is allowed. In this paper, a bicluster with a subset of rows *R *and a subset of columns *C *is denoted by (*R*, *C*). At the beginning, the first-level difference matrix D_1 _is calculated for the input expression matrix E as described in line 4 in Figure 7. Supposed that E has size *m *rows by *n *columns. There is altogether *n*(*n *- 1)/2 different number of permutations so the size of D_1 _is *m *× *n*(*n *- 1)/2. In order to derive possible biclusters, a simple clustering algorithm can be applied to identify clusters for each column (lines 6–12). Let *X *= {*x*_1_, *x*_2_,...,*x*_*N*_} be a set of *N *expression values. By comparing *x*_*i *_with all values in *X*, a set of values *S*_*i *_similar to *x*_*i *_can be found as follows,

(1) *S*_*i *_= {*a *∈ *X *:|*x*_*i *_- *a*| <*ε*}

where *i *= 1, 2, ..., *N*. Also, the set of indices *Q*_*i *_associated with the values in *S*_*i *_can be obtained. *Q*_*i *_can be expressed by

(2) *Q*_*i *_= {*p *∈ {1, 2, ..., *N*}: *x*_*p *_∈ *S*_*i*_}

As an example, given that *X *= {1, 2, 9, 3} and *ε *= 2. *S*_2 _= {1, 2, 3} and *Q*_2 _= {1, 2, 4}. A clustering algorithm based on equation (1) would generate *N *clusters but these clusters may be very close to each other and have large overlap. In order to reduce unnecessary clusters, we adopt a two-step clustering approach presented in lines 56–84. In addition to the definitions in (1) and (2), let us denote the current collections of clusters and corresponding sets of indices by *S *and *Q*, which are both set to be empty initially. In the first step (lines 58–75), for *i *= 1, 2, ..., *N*, *x*_*i *_and its associated cluster *S*_*i *_are tested for the following three conditions with each *S*_*j *_∈ *S*:

(1) |*x*_*i *_- ${\bar{S}}_{j}$| ≥ *ε *where $\bar{•}$ denotes the average operation of a set.

(2) |*S*_*i*_| ≥ *N*_*r*_, where |•| denotes the cardinality of a set.

(3) $|{\bar{S}}_{i}-{\bar{S}}_{j}|\geq \varepsilon$.

If the above three conditions are satisfied for all *S*_*j *_∈ *S*, *S*_*i *_and *Q*_*i *_are added to *S *and *Q *respectively. In the second step (lines 76–82), the clusters are refined. Denote the sets of output clusters and the corresponding indices by *S*' and *Q*' respectively. First, *S*' and *Q*' are set to be empty. For each *S*_*j *_∈ *S*, a new cluster *S*'_*j *_is derived as

(3) $$S{'}_{j}=\{a\in X:\left|a-{\bar{S}}_{j}\right|<\varepsilon \}$$

The corresponding set of indices *Q*'_*j *_is given by

(4) *Q*'_*j *_= {*p *∈ {1, 2, ..., *N*}:*x*_*p *_∈ *S*'_*j*_}

*S*'_*j *_and *Q*'_*j *_are added to *S*' and *Q*' respectively if |*S*_*j*_'| ≥ *N*_*r*_. For the first-level difference matrix D_1_, each *Q*'_*j *_contains the row indices of the cluster *S*'_*j*_. Each column of D_1 _consists of difference values between column *i *and *j *of the original expression matrix. Define the collection of row indices sets of the clusters to be *U*_*ij*_. After finding all *U*_*ij *_for all distinct column pairs (*i*, *j*), the row indices set of the clusters and their associated column pairs are collected to form a list of possible biclusters *L*_1 _which can be expressed by

(5) *L*_1 _= {(*R*_*ij*_, (*i*, *j*)): *U*_*ij *_≠ *φ*, *R*_*ij *_∈ *U*_*ij*_, *i *= 1, 2, ..., *n *- 1 and *j *= *i *+ 1, *i *+ 2,...,*n*}

As one always tries to find the biggest bicluster, a sorting is performed for the possible biclusters in *L*_1 _based on the number of rows in line 13 so that a bicluster with the largest number of rows can be processed first.

Starting from the biggest bicluster *l*_1 _in the sorted list of possible biclusters *L*_2_, the second-level difference matrix D_2 _is formed as in line 24 in which one of the bicluster columns (column *c*_*k*1 _or *c*_*k*2_) is compared with all the remaining columns on those chosen rows (e.g. difference matrices illustrated in Figure 3). Note that the second-level difference matrix D_2 _can be obtained directly from the first-level difference matrix D_1_. Before D_2 _calculation, early termination can be introduced as presented in lines 17–23 as an optional step. In the early termination, the biclusters in *L*_2 _which significantly overlap with the identified biclusters are skipped as they are unlikely to derive a well-distinguishable bicluster according to the given parameter *P*_*o*_. Similar to the clustering done for D_1_, clustering and sorting are performed for D_2 _as described in lines 26–33. As a result, a list of possible column segments *H*_2 _for growing the current bicluster is obtained. In lines 34–41, a possible bicluster (*R*, *C*) is constructed based on the row intersection with each column segment in *H*_2_. Initially, (*R*, *C*) is set to be the current bicluster *l*_*k *_in *L*_2_. If the size of the row set *R *does not fall below the user-defined threshold *N*_*r *_after the row intersects with a column segment, the column is included in *C *and *R *is updated. Otherwise, the process is moved to the next column segments until the last one is examined. Finally, the bicluster is validated with respect to the given requirements in bicluster size and degree of overlap as depicted in lines 42–50. Only a valid bicluster is output (lines 51–53).

### Relation to existing *δ*-pCluster approaches

The proposed algorithm identifies biclusters which are homogeneous in each column pair. In this section, we show that the biclusters can be expressed as *δ*-pClusters [25]. Hence, any sub-matrix in an identified bicluster has similar homogeneity to that bicluster and the problem of outliers as in Cheng and Church algorithm [12] can be avoided. Denote a bicluster with a subset of rows *U *and a subset of columns *V *by *B *= (*U*, *V*). The bicluster *B *is a *δ*-pCluster if for each 2 × 2 sub-matrix *M*, the following condition holds

(6) |*a*_*ij *_- *a*_*in *_- (*a*_*mj *_- *a*_*mn*_)| ≤ *δ*

where $M=\left[\begin{array}{cc} a_{ij} & a_{in}\\ a_{mj} & a_{mn} \end{array}\right]$, *a*_*ij *_denotes a value of the expression matrix at position (*i*, *j*), *i*, *m *∈ *U *and *j*, *n *∈ *V*. In our algorithm, the clustering (the second step) performed in the second-level difference matrix ensures that there exists a column *k *∈ *V *such that

(7) |*a*_*ij *_- *a*_*ik *_-*l*_*jk*_| <*ε *for ∀*j *∈ *V *and some constant *I*_*jk*_

where *ε *is the noise threshold parameter of the proposed algorithm. Hence, for any *i*, *m *∈ *U*, we have

(8) |*a*_*ij *_- *a*_*ik *_- (*a*_*mj *_- *a*_*mk*_)| = |*a*_*ij *_- *a*_*ik *_- *l*_*jk *_- (*a*_*mj *_- *a*_*mk *_- *l*_*jk*_)| ≤ |*a*_*ij *_- *a*_*ik *_- *l*_*jk*_| + |*a*_*mj *_- *a*_*mk *_- *l*_*jk*_| < 2*ε*

where the last inequality follows from inequality (7). For a column *n *∈ *V *with *n *≠ *k*, using inequality (8), it is shown that

(9) |*a*_*ij *_- *a*_*in *_- (*a*_*mj *_- *a*_*mn*_)| = |*a*_*ij *_- *a*_*ik *_- (*a*_*mj *_- *a*_*mk*_) + *a*_*ik *_- *a*_*in *_- (*a*_*mk *_- *a*_*mn*_)| ≤ |*a*_*ij *_- *a*_*ik *_- (*a*_*mj *_- *a*_*mk*_)| + |(*a*_*ik *_- *a*_*in*_) - (*a*_*mk *_- *a*_*mn*_)| < 4*ε*

This means that the bicluster *B *is a *δ*-pCluster with *δ *= 4*ε*. Although the biclusters identified by our algorithm are *δ*-pClusters, it should be emphasized that our algorithm is not designed specially for detecting *δ*-pClusters but rather is based on the clustering results in the difference matrix. Hence, there are some differences between our biclustering strategy and the other *δ*-pClusters algorithms like pCluster algorithm [25] and S. Yoon et al. approach [13]. Specifically, our algorithm takes into account the cluster density in which cluster centroids are considered. In contrast, the other two *δ*-pCluster based algorithms rely only on the inter-distances between elements in the difference matrix as defined by the inequality (6). This results in an exponential-time complexity in the worst case. Our proposed algorithm can be regarded as a greedy version of the other two algorithms. In particular, for each column-pair bicluster, our proposed algorithm derives a possible bicluster by greedily finding a larger column set through sequential intersection with other column-pair biclusters. The large column-pair biclusters usually contain the whole or a large part of the true gene set. On the other hand, these simplifications significantly reduce the complexity from exponential-time to polynomial-time.

### Complexity estimation

In general, a biclustering problem is NP-complete [11]. However, we have adopted a simple clustering algorithm and bicluster growing strategy to reduce the complexity. Given a matrix of size *m *× *n*, the complexity of obtaining the difference matrix is *O*(*mn*^2^). The simple clustering algorithm applied on each column requires operations on the order of *O*(*m*^2^) because it involves comparing the value of each element with the others and the centroids of the found clusters. In addition, the total number of clusters found would not exceed *m*. Therefore, the complexity in obtaining clusters in the difference matrix is *O*(*m*^2^*n*^2^) and the number of clusters is at most *mn*(*n*-1)/2. The sorting of the clusters requires a complexity of *O*(*mn*^2 ^log *mn*^2^). After that, each identified cluster is used as a seed to construct a bicluster. In the biclusters growing process, a seed is first checked if it has significant overlap with other identified biclusters for early termination. The overlapping in rows can be checked by sorting followed by element-wise comparison. The complexity is thus *O*(*m*log*m*). For columns, as a seed has only two columns, the complexity is *O*(*n*). Note that the number of identified biclusters is bounded by the number of seeds. Thus, the complexity for checking overlaps in all identified biclusters is *O*(*mn*^2 ^(*n *+ *m *log *m*)). If the seed is valid, a sub-matrix of the difference matrix is extracted as the second-level difference matrix. This step requires no arithmetic operations due to data reuse. Clustering and sorting procedure are then performed on this second-level difference matrix. As the matrix has *n*-1 columns only, the clustering and the sorting processes need operations on the order of *O*(*m*^2^*n*) and *O*(*mn *log(*mn*)), respectively. Note that there are at most (*n*-1)*m *clusters detected in the second-level difference matrix. In the bicluster construction, row intersection is performed. In total, the complexity is *O*(*m*^2^*n *log *m*). Finally, the new identified bicluster is validated (i.e. filtered) with respect to the number of columns and degree of overlap with other biclusters. The validation requires an additional complexity of *O*(*mn*^2 ^(*m *log *m *+ *n *log *n*)). Among the operations for obtaining each biclusters from the first-level difference matrix, the validation step dominates. So the entire processing for bicluster formation from seeds is *O*(*m*^2^*n*^4 ^(*m *log *m *+ *n *log *n*)). Since this cost dominates all other costs in previous steps, our algorithm has a polynomial-time complexity of *O*(*m*^2^*n*^4 ^(*m *log *m *+ *n *log *n*)). The above estimation shows the worst case complexity, in which the validation process dominates. In practice, the number of biclusters is far less than *mn*(*n *- 1)/2. Moreover, some of the validation steps can be avoided through early termination of invalid biclusters. Elimination of invalid biclusters reduces the number of potential biclusters and this in turn reduces the complexity inside the validation step.

### Modification for multiplicative models

As seen in Figure 1(E), a multiplicative-related bicluster is a bicluster in which any two rows are related by the same ratio in all the related columns or any two columns are related by the same ratio in all the related rows. In order to modify the proposed framework for multiplicative models, the difference matrix is replaced by a ratio matrix which is in the form of *c*_*i*_/*c*_*j *_or *c*_*j*_/*c*_*i *_for all the *n*(*n *- 1)/2 distinct combinations between columns *i *and *j *where *c*_*k *_represents the values in the *k*-th column. In practice, we select the column which has the largest average magnitude as the denominator because quotient is sensitive to noise when the divisor is small. Thus, the major change for detecting multiplicative-related biclusters is to replace the difference matrix by a ratio matrix. Note that the complexity for multiplicative models is essentially the same as that for additive models.

### Interactive adjustment of noise threshold using PC plots

**Figure 8:**
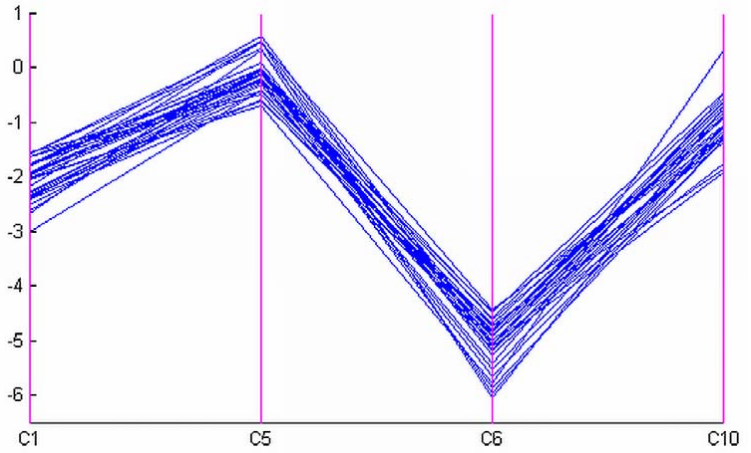
The PC plot of the true additive-related bicluster.

**Figure 9:**
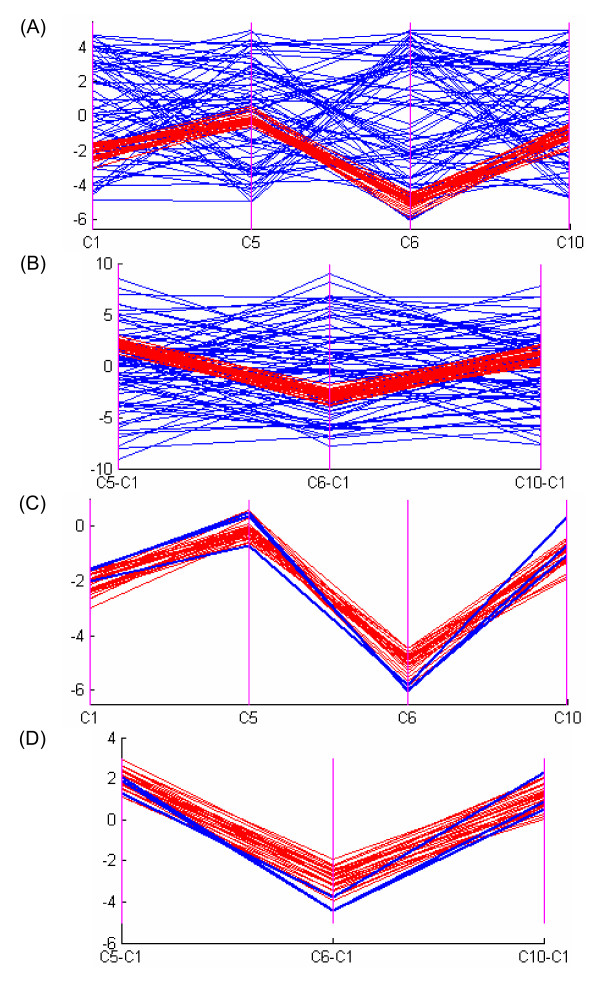
**The PC plots of the bicluster identified using noise threshold of 1.2**. The expression values of all rows in the four related columns and their difference between the last three columns and the first column are drawn using PC plots in Figures (A) and (B) respectively. In Figures (A) and (B), red color shows rows from the identified bicluster while blue color shows rows from the original dataset. Figures (C) and (D) illustrate the inconsistencies between the identified bicluster and the true bicluster in the four related columns and the column differences respectively. In Figures (C) and (D), the red color indicates rows of the true bicluster that are found by our algorithm while the blue color represents the three missed rows of the true bicluster.

The setting of the noise threshold *ε *is important for the proposed algorithm as it balances the homogeneity requirement and the noise tolerance in the identified biclusters. The noise threshold is determined through visual inspection of the homogeneity of the detected biclusters in the PC plots [26]. The PC visualization for a data matrix embedded with biclusters can be found in Additional file 1. Consider a noisy 100 × 10 dataset which contains uniformly distributed values between -5 and 5 embedded with a 30 × 4 additive-related bicluster shown in Figure 8. Furthermore, an additive Gaussian noise with variance of 0.2, which was chosen empirically for clear demonstration, was introduced. In this example, we varied the values of the noise threshold *ε *while fixing the values of the minimum number of rows *N*_*r*_, the minimum number of columns *N*_*c *_and the maximum overlap with other biclusters *P*_*o *_to be 20, 4 and 20%, respectively. Figure 9 shows a bicluster found by our algorithm when *ε *is set to 1.2. The four columns are found correctly, however, three rows are missed. Figure 9(A) shows the four columns but with all the rows in the original data while Figure 9(B) shows the difference between the last three columns with respect to the first column. Figures 9(C) and 9(D) illustrate the inconsistency between the identified bicluster and the true bicluster using PC plots of expression values and difference values respectively in which the three missed rows are displayed in blue. We can see that these three rows are missed because the noise threshold is not large enough. In practice, since we do not know the bicluster in advance, we should adopt an exploratory approach for setting the parameter *ε*. Start with the current value of *ε*, we gradually increase *ε *while visualizing the bicluster using the PC plot. Initially, we would see more and more related rows being included into the bicluster. Then, at some point, unrelated rows start to creep into the bicluster. When this is observed in the PC plot, we stop increasing the noise threshold. Using this procedure, we found that when *ε *is set to 1.5, all the rows are correctly detected. This example shows that the PC plot can be a powerful visualization and interactive tool that allows us to examine the biclusters found.

## Results and Discussion

### Evaluation methods

We analyze the performance of our algorithm on both artificial datasets and a real dataset. For artificial datasets, biclusters information is known in advance. So accuracy in bicluster discovery can be measured using the overall match score [16]. The overall match score of a set of biclusters *M*_1 _with respect to another set of biclusters *M*_2 _is defined as,

(10) $$S^{*}(M_{1},M_{2})=\sqrt{S_{U}^{*}(M_{1},M_{2})\times S_{V}^{*}(M_{1},M_{2})}$$

where $S_{U}^{*}(M_{1},M_{2})$ and $S_{V}^{*}(M_{1},M_{2})$ are gene and condition match scores respectively. $S_{U}^{*}(M_{1},M_{2})$ is calculated as,

(11) $$S_{U}^{*}(M_{1},M_{2})=\frac{1}{\left|M_{1}\right|}\sum_{(U_{1},V_{1})\in M_{1}}\underset{(U_{2},V_{2})\in M_{2}}{\max}\frac{\left|U_{1}\cap U_{2}\right|}{\left|U_{1}\cup U_{2}\right|}$$

where a bicluster with a subset of genes *U*_*i *_and a subset of conditions *V*_*i *_is denoted by (*U*_*i*_, *V*_*i*_). $S_{V}^{*}(M_{1},M_{2})$ is defined similarly with *U *replaced by *V*. Let *M *be the set of detected biclusters and *M*_*t *_be the set of true biclusters embedded in the artificial expression dataset. The overall match score S*(*M*, *M*_*t*_) quantifies the average relevance of the detected biclusters to the true biclusters. Conversely, S*(*M*_*t*_, *M*) measures the average recovery of the true biclusters in the detected biclusters. To unify the two measures into a single quantity for evaluation, their average is computed as the biclustering accuracy.

The performance of the proposed algorithm for artificial datasets has been compared with two existing algorithms with the additive model assumption, namely the Cheng and Church (C&C) algorithm [12] and the pCluster algorithm [25]. We considered the biclustering accuracy together with other measures such as number of biclusters, bicluster size and processing time. The programs for both algorithms are publicly available [27,28]. The proposed algorithm was implemented in a C MEX-file and ran in Matlab 6.5. All the experiments were conducted on the Window XP platform in a computer with 2.4 GHz Intel Pentium 4 CPU and 512 MB RAM. In identification of multiplicative-related biclusters, since C&C algorithm and the pCluster algorithm are designed for additive models, logarithm operation was applied to the expression data so that the multiplicative models become additive models. For comparison, we also applied the proposed algorithm for additive models to the logarithm values. Henceforth, the proposed algorithm for additive models and multiplicative models will be referred to as PA and PM respectively while the proposed algorithm for additive models with the logarithm operation as pre-processing will be referred to as PAL.

The evaluation on real datasets was performed on three aspects: biological, homogeneity and statistical assessment. In the biological assessment, we used the Gene Ontology (GO) annotations [29] to determine the functional enrichment of biclusters. The measure was the percentage of overrepresented biclusters in one or more GO annotation. A bicluster is said to be overrepresented in a functional category if it gives a small *p*-value. Given that a bicluster *B *with *k *genes is identified in a gene expression matrix with a gene set *S *of size *N*. For a functional category with *C *genes in *S*, the bicluster *B *possesses *r *genes. The *p*-value is defined as the probability of choosing *k *genes from *S *with *r *genes in that category [30], i.e.,

(12) $$p-value(B)=\left(\begin{array}{c} C\\ r \end{array}\right)\left(\begin{array}{c} N-C\\ k-r \end{array}\right)/\left(\begin{array}{c} N\\ k \end{array}\right)$$

In other words, the *p*-value is the probability of including genes of a given category in a cluster by chance. Thus, the overrepresented bicluster is a cluster of genes which is very unlikely to be obtained randomly. The annotations consist of three ontologies, namely biological process, cellular component and molecular function.

For the homogeneity aspect, mean squared residue score (MSRS) [12] and average correlation value (ACV) [31] were computed. For an *m *× *n *bicluster, the MSRS is defined as

(13) $$\text{MSRS}=\frac{1}{mn}\sum_{i=1}^{m}\sum_{j=1}^{n}{\left(a_{ij}-{\tilde{a}}_{i•}-{\tilde{a}}_{•j}+\tilde{a}\right)}^{2}$$

where *a*_*ij *_is the value of the bicluster at position (*i*, *j*), ${\tilde{a}}_{i•}$ is the average of the *i*-th row, ${\tilde{a}}_{•j}$ is the average of the *j*-th column and $\tilde{a}$ is the overall average. ACV is defined by

(14) $$\text{ACV}=\max\{\frac{\sum_{i=1}^{m}\sum_{j=1}^{m}\left|c\_row_{ij}\right|-m}{m^{2}-m},\frac{\sum_{i=1}^{n}\sum_{j=1}^{n}\left|c\_col_{ij}\right|-n}{n^{2}-n}\}$$

where *c*_*row*_*ij *_is the correlation coefficient between rows *i *and *j *and *c*_*col*_*pq *_is the correlation coefficient between columns *p *and *q*. ACV is applicable to additive models as well as multiplicative models but the MSRS is valid only for additive models. In order to measure homogeneity of multiplicative-related biclusters, logarithm was applied onto the expression values before calculating MSRS values so that a multiplicative-related bicluster can be formulated using an additive model. In order to avoid confusion, the MSRS for the logarithm of expression values is denoted by MSRS_l_. A bicluster with high homogeneity in expression levels should have a low MSRS/MSRS_l _value but a high ACV value. The minimum value of MSRS/MSRS_l _is zero while ACV has a maximum value of one.

The statistical properties of the biclustering results refer to quantities including the number of discovered biclusters and the bicluster size. Comparative studies were performed in the three aspects with several existing biclustering algorithms such as C&C, iterative signature algorithm (ISA) [32,33], order-preserving submatrix (OPSM) approach [1] and xMotifs [34], which are available in [27]. In addition, the computational complexity of the proposed algorithm and other approaches is estimated using processing time as done for the artificial datasets. Despite the dependence of factors such as programming language and parameter settings, a rough comparison in complexity can still be achieved.

### Datasets

Two types of artificial datasets were considered, one for the additive models and the other for the multiplicative models. The first type of dataset TD1 had a size of 200 rows by 40 columns. Uniformly distributed random values were first generated. Then four biclusters were embedded. Their details are as follows:

• bicluster A is a constant row bicluster of size 40 × 7;

• bicluster B is a constant row bicluster of size 25 × 10;

• bicluster C is a constant column bicluster of size 35 × 8; and

• bicluster D has coherent values related by additions of size 40 × 8.

**Figure 10:**
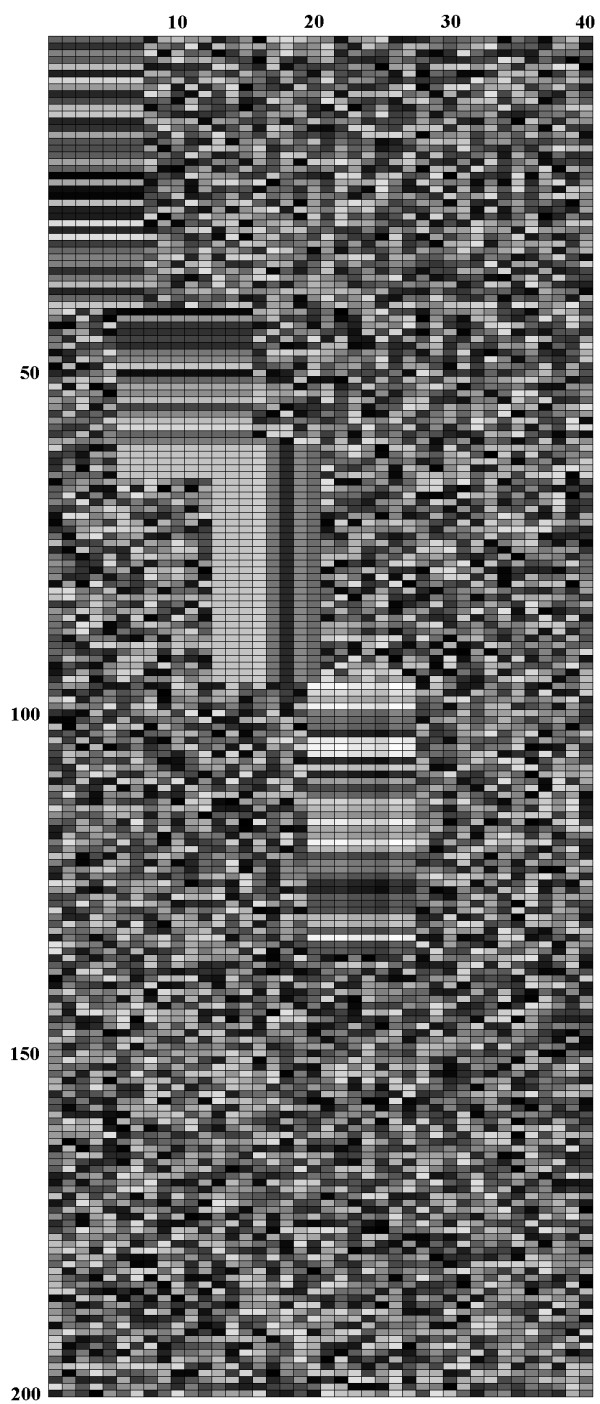
The first type of dataset with four additive-related biclusters before noise is added.

Biclusters A and B have two columns in common but in different rows; bicluster B overlaps with bicluster C in five rows and three columns; biclusters C and D have one column in common but in different rows. Finally, Gaussian noise with different standard deviation (s.d.) was added to the dataset. At each non-zero noise level, five expression matrices were generated. Figure 10 shows the dataset TD1 with 4 embedded biclusters before noise was added.

**Figure 11:**
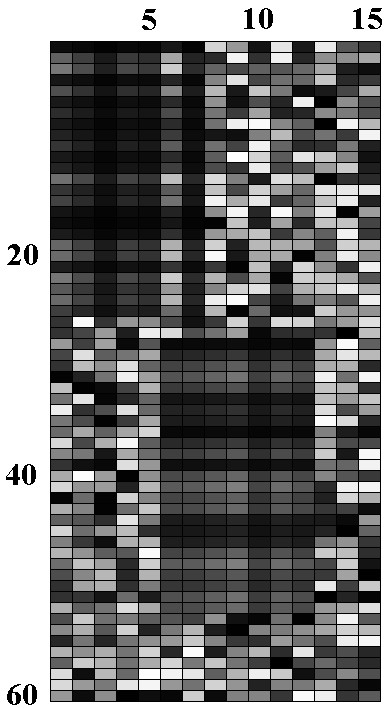
The second type of dataset with two multiplicative-related biclusters before noise is added.

The second type of dataset TD2 consists of 60 × 15 positive values embedded with two 25 × 7 multiplicative-related biclusters. The two biclusters overlap in two columns. A positive-biased Gaussian noise was added to the dataset so that all the values in the resultant datasets remained positive. The positive-valued dataset was essential for Cheng and Church algorithm, the pCluster algorithm and our proposed algorithm for the additive models PAL due to the use of the logarithm operation. It should be noted that the proposed algorithm for multiplicative-related biclusters PM can be applied on datasets with negative values because no logarithm operation is needed. Figure 11 shows the dataset TD2 with two embedded multiplicative biclusters before noise was added. These two artificial datasets allowed us to test the performance of our algorithm in realistic situations as real expression data often involves various types of biclusters with overlaps (i.e. regulatory complexity) and noise.

The real dataset used was the yeast Saccharomyces cerevisiae cell cycle dataset as used in [12], which contains 2884 genes and 17 conditions. The non-missing values were all non-negative. As multiplicative models were also investigated, those zero non-missing values were set to some small positive values. The missing values were filled with positive uniformly distributed random values to minimize the influence to our analysis.

### Performance on artificial datasets

**Figure 12:**
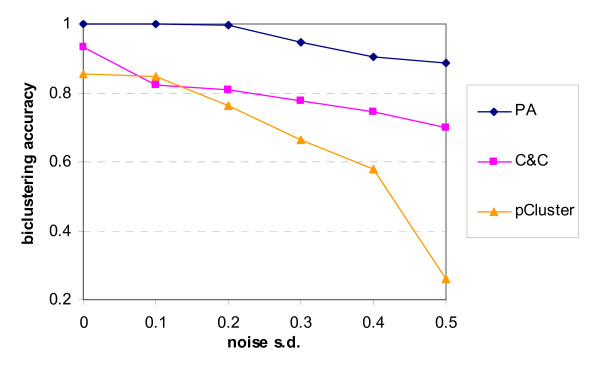
**Biclustering accuracy against noise level for additive models**. The biclustering accuracies of the proposed algorithm, Cheng and Church algorithm and pCluster algorithm are represented by the curves 'Proposed', 'C&C' and 'pCluster' respectively.

For the artificial datasets with additive-related biclusters, biclusters with rows and columns more than or equal to 21 and 5 respectively were identified. It was further required that any detected bicluster cannot have more than 50% overlap with another bicluster simultaneously in the row and column dimensions. Since the Cheng and Church (C&C) algorithm and the pCluster algorithm cannot be directly configured to discover biclusters with all the given requirements, a post-filtering procedure was adopted to eliminate those invalid biclusters. The post-filtering parameters are provided in Table 1 together with the parameters of the biclustering algorithms. Note that parameters for noise tolerance (*ε*/*δ*) were determined for optimal performance under different noise levels. The biclustering accuracies are plotted against various noise levels in Figure 12. As can be seen, the proposed algorithm always has higher biclustering accuracy than C&C and the pCluster algorithm. For the expression dataset with noise of standard deviation at or below 0.1, we detect the four embedded biclusters perfectly. The pCluster algorithm did not attain perfect discovery even in the noise-free case because more than one maximal *δ*-pCluster (defined by equation (6)) exists for one or more column pair due to column overlap between some biclusters in the datasets [35]. In more noisy case such as when the noise s.d. is 0.5, the biclustering accuracy of our algorithm still has a high value of 0.89. In contrast, the accuracies of C&C and the pCluster algorithm are 0.70 and 0.26 respectively.

**Table 1:** Parameter settings for biclustering algorithms and post-filtering in the experiments on artificial datasets

Experiment	Algorithm/post-filtering	Parameter settings*
Artificial datasets for additive models	PA	*ε *= 0.5 – 2.0, *N*_*r *_= 21, *N*_*c *_= 5, *P*_*o *_= 50
	C&C	*δ *= 0.04 – 0.5, α = 1.2, M = 40
	pCluster	*δ *= 0.5 – 1.0, *N*_*r *_= 21, *N*_*c *_= 5
	Post-filtering	*N*_*r *_= 21, *N*_*c *_= 5, *P*_*o *_= 50 and M = 10
Artificial datasets for multiplicative models	PM	*ε *= 0.2 – 0.6, *N*_*r *_= 18, *N*_*c *_= 4, *P*_*o *_= 25
	PAL	*ε *= 0.4 – 1.0, *N*_*r *_= 18, *N*_*c *_= 4, *P*_*o *_= 25
	C&C	*δ *= 0.04 – 0.5, α = 1.2, M = 20
	pCluster	*δ *= 0.5 – 1.0, *N*_*r *_= 18, *N*_*c *_= 4
	Post-filtering	*N*_*r *_= 18, *N*_*c *_= 4, *P*_*o *_= 25 and M = 5

* The definitions of parameters *ε*, *N*_*r*_, *N*_*c *_and *P*_*o *_follow those defined for the proposed algorithm, i.e. noise threshold, minimum number of rows, minimum number of columns and maximum percentage in overlap allowed in biclusters respectively. Furthermore, M denotes the maximum number of biclusters required and *δ *of C&C and the pCluster algorithm is defined as in the original publications [12, 25].

Statistical properties of the biclustering results before filtering are given in Table 2. Unlike the pCluster algorithm, the number of biclusters identified by the proposed algorithm is insensitive to noise level. On average, there were 6.6 biclusters identified at the highest noise level which was close to the true number 4. For the pCluster algorithm, a large number of biclusters with high overlap were detected under noisy situation. The post-filtering procedure was therefore necessary for the pCluster algorithm to extract the significant biclusters. The number of biclusters identified by C&C was 40 which is the same as that specified in its parameter setting. In fact, this parameter setting was necessary to acquire high biclustering accuracy. With respect to the biclusters size, the proposed algorithm shows the closest agreement to those embedded in the datasets. The average numbers of rows and columns in the biclustering results are always around 34 and 7.8 respectively while the actual average numbers of rows and columns are 35 and 8.25 respectively. The pCluster algorithm also produced good results. C&C gave the worst performance as it does not allow any constraints to be imposed on the biclusters dimensions. Therefore, the post-filtering procedure is essential for C&C to find the embedded biclusters.

**Table 2:** Statistical properties of biclustering results for the artificial datasets embedded with additive-related biclusters before post-filtering

Property	Algorithm	Noise s.d.					
		
		0	0.1	0.2	0.3	0.4	0.5
Average number of biclusters	PA	4	4	4	4.4	5.8	6.6
	C&C	40	40	40	40	40	40
	pCluster	23	366.6	378.2	255.6	124.2	21.40
Average number of rows	PA	35	35	34.85	33.67	31.87	32.25
	C&C	7.625	7.640	8.025	7.980	8.265	7.895
	pCluster	25.57	23.14	23.28	22.98	22.69	21.74
Average number of columns	PA	8.250	8.250	8.250	7.480	7.262	7.228
	C&C	3.725	4.500	4.915	4.765	5.070	5.280
	pCluster	5.217	5.224	5.115	4.873	4.468	4.237

**Figure 13:**
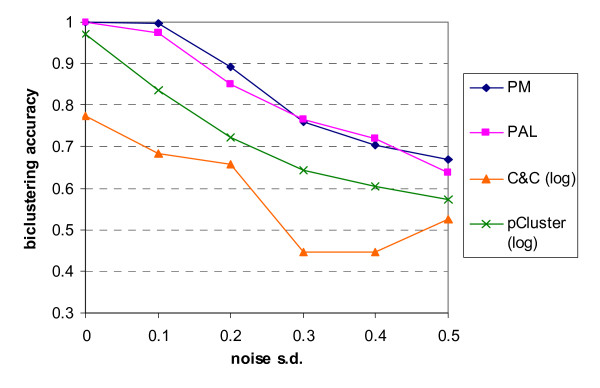
**Biclustering accuracy against noise level for multiplicative models**. The biclustering accuracy of the proposed algorithms for multiplicative models is denoted by 'PM' while the proposed algorithms for additive models, Cheng and Church algorithm and pCluster algorithm on logarithm of expression data are labelled by 'PAL', 'C&C (log)' and 'pCluster (log)' respectively.

For the datasets with two multiplicative-related biclusters, a bicluster was considered to be valid if its size is no smaller than 18 and 4 in row and column dimensions respectively and the overlap with other valid biclusters is less than or equal to 25%. The settings for the biclustering algorithms and the post-filtering procedure are also included in Table 1. The biclustering accuracies of the proposed algorithms PAL and PM, together with C&C and the pCluster algorithm (applied on log values) at various noise levels is shown in Figure 13. At all the noise levels, our two proposed algorithms outperform C&C and the pCluster algorithm. Both PAL and PM can exactly detect the true biclusters in the noise-free case while the other two algorithms fail to do so. In particular, the failure of perfect discovery in the pCluster algorithm can be attributed to the column overlap in the datasets. The performance of PM is slightly better than that of PAL in general. The biclustering accuracy decreases when the noise level increases except in the case of C&C when noise level changes from 0.4 to 0.5. It was probably because outlier is less likely to be included in biclusters at high noise levels. In terms of the statistical properties given in Table 3, the two proposed algorithms exhibit closest match to the true embedded biclusters, with PM performs slightly better than PAL. Similar to the case of the additive models, the proposed algorithms can return more reasonable number of biclusters with similar dimensions to those embedded than the other two algorithms without any post-filtering procedure.

**Table 3:** Statistical properties of biclustering results for the artificial datasets embedded with multiplicative-related biclusters before post-filtering

Property	Algorithm	Noise s.d.					
		
		0	0.1	0.2	0.3	0.4	0.5
Average number of biclusters	PM	2	2	2.2	3	2.4	2.6
	PAL	2	2	2.2	3.2	3	3.6
	C&C	20	20	20	20	20	20
	pCluster	1109	956.4	855.8	753.4	658.8	729.2
Average number of Rows	PM	25	24.80	24.17	23.72	20.20	23.40
	PAL	25	23.70	21	20.10	19.52	19.72
	C&C	5.850	5.560	6.900	8.820	8.270	6.940
	pCluster	19.94	19.93	19.87	19.75	19.71	19.66
Average number of columns	PM	7	7	6.367	6.400	5.900	5.933
	PAL	7	7	6.433	5.517	5.567	5.417
	C&C	4.300	3.870	5.020	5.660	5.570	5.110
	pCluster	4.346	4.345	4.297	4.224	4.178	4.158

In order to justify the efficiency of our proposed algorithms, processing time for the artificial datasets with noise s.d. of 0.3 was measured and provided in Table 4. The proposed algorithm PA required an average of 3.8 sec for the artificial datasets with additive-related biclusters. This showed substantial improvement over the pCluster algorithm which needed 2716 sec to finish. The reduction in computational complexity is achieved by the bicluster growing strategy in which similar patterns in column-pair are combined to form biclusters through row intersection. The proposed algorithm is also more efficient than C&C by a factor of 4.5. For datasets embedded with multiplicative-related biclusters, the matrices sizes are smaller than those used for additive-model experiments so less processing time was obtained in all the algorithms. However, it can be seen that the proposed approach PM has the lowest computational complexity. The average processing time was 0.0232, 1 and 105.2 sec for PM, C&C and the pCluster algorithm respectively. In conclusion, the results on artificial datasets demonstrate that our proposed algorithms have high accuracy in detecting additive-related and multiplicative-related biclusters, even in the presence of overlap and noise contamination. The computational complexity of the proposed algorithms is lower than several biclustering algorithms with similar model assumption.

**Table 4:** Average processing time for the artificial datasets

Dataset	Artificial datasets for additive models with noise s.d. of 0.3			Artificial datasets for multiplicative models with noise s.d. of 0.3		
		
Algorithm	PA	C&C	pCluster	PM	C&C (log)	pCluster (log)
		
Average time (sec)	3.776	17	2716	0.0232	1	105.2

### Performance on a real dataset

**Figure 14:**
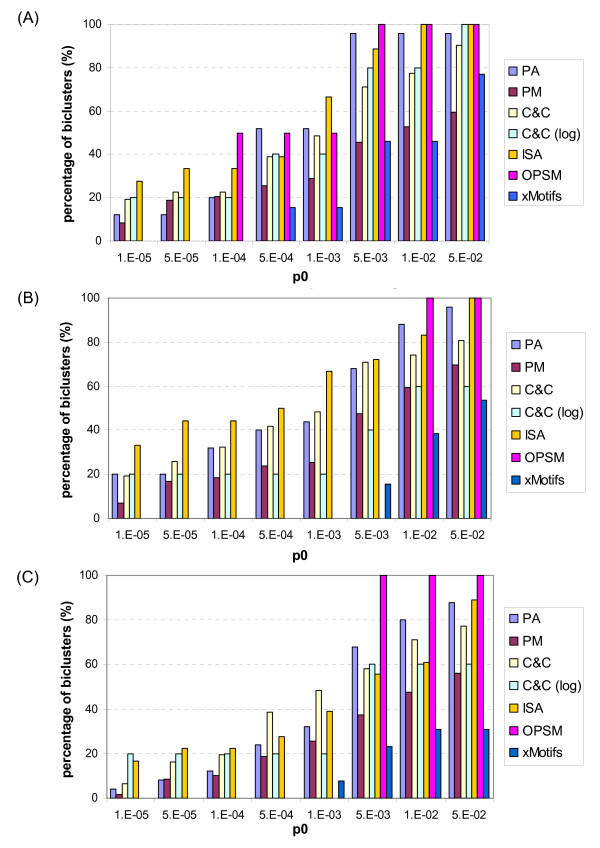
**Percentage of additive-related biclusters enriched with GO annotations of different ontologies**. (A) Biological process ontology. (B) Cellular component ontology. (C) Molecular function ontology.

Experiments have been conducted on the yeast cell cycle dataset using the proposed algorithms and Cheng and Church (C&C) algorithm [12], iterative signature algorithm (ISA) [32,33], order-preserving submatrix (OPSM) approach [1] and xMotifs [34]. Post-filtering was applied to the biclustering results in order to eliminate insignificant biclusters as well as impose common constraints for comparison. The parameter settings of various algorithms and post-filtering are provided in Table 5. These values were selected based on the guideline in [16] and our experimental work. The functional enrichment was studied over a number of upper bounds on *p*-value, *p*_0 _and illustrated in Figure 14. Compared with C&C which possesses the same model assumption as the proposed algorithm for additive model (PA), higher percentage of functionally-enriched biclusters were identified by the proposed algorithm at *p*_0 _≥ 5 × 10^-4^, 1 × 10^-2 ^and 5 × 10^-3 ^in the biological process, cellular component and molecular function ontologies respectively. In particular, at *p*_0 _= 1 × 10^-2^, the percentage of functionally-enriched biclusters found by PA is 96.0%, 88.0% and 80.0% which correspond to an improvement of 18.6%, 13.8% and 9.0% to C&C in the biological process, cellular component and molecular function ontologies, respectively. At the lowest value of *p*_0 _= 1 × 10^-5^, our proposed algorithm PA outperforms C&C in the cellular component ontology but not in the other two ontologies. However, the reduction in the percentage of functionally-enriched biclusters is less than 7.5% in the biological process ontology and 2.5% in the molecular function ontology, which is relatively small compared with the improvement at the large values of *p*_0_. The homogeneous analysis provided in Table 6 shows that the biclusters identified by PA are more homogeneous than C&C with the average MSRS lower by 142.4 and the average ACV higher by 0.0199. From the statistical results in Table 7, it can be found that PA can also avoid identification of very large bicluster as is in the case of C&C. The largest bicluster size found using PA is 597 × 17 while that found using C&C is 1391 × 17.

**Table 5:** Parameter settings of algorithms and post-processing investigated in experiments based on the yeast dataset

Algorithm/post-filtering	Parameter settings*
PA	*ε *= 60, *N*_*r *_= 10, *N*_*c *_= 5, *P*_*o *_= 20
PM	*ε *= 0.2, *N*_*r *_= 10, *N*_*c *_= 5, *P*_*o *_= 20
C&C	*δ *= 100, *α *= 1.2, *M *= 100
C&C (log)	*δ *= 0.25, *α *= 1.2, *M *= 100
ISA	*t*_*g *_= 2, *t*_*c *_= 1.0, number of initial sets = 500
OPSM	*l *= 100
xMotifs	*n*_*s *_= 10, *n*_*d *_= 1000, *s*_*d *_= 4, *p*-value = 10^-10^, *α *= 0.29, max. number of expression values = 50
Filtering	*N*_*r *_= 10, *N*_*c *_= 5, *P*_*o *_= 20

* The definitions of parameters *ε*, *N*_*r*_, *N*_*c*_, *P*_*o *_and *M *follow those defined in the experiments on artificial datasets. The other parameters in C&C, ISA, OPSM and xMotifs are as defined in their original publications [1, 12, 32-34].

**Table 6:** Homogeneity comparison of biclusters identified in the yeast cell-cycle dataset using various algorithms

Algorithm	MSRS/MSRS_l_*			ACV		
		
	min	mean	max	min	mean	max
PA	326.0	412.1	552.7	0.8960	0.9416	0.9755
PM	3.694 × 10^-4^	9.573 × 10^-3^	3.809 × 10^-2^	0.7493	0.9219	1
C&C	439.6	554.5	593.3	0.6481	0.9217	0.9768
C&C (log)	2.784 × 10^-2^	6.262 × 10^-2^	8.451 × 10^-2^	0.3489	0.5740	0.9000
ISA	108.9	489.6	794.6	0.8420	0.9247	0.9588
OPSM	480.4	497.1	513.8	0.8866	0.8904	0.8941
xMotifs	1.910 × 10^-12^	4.820	12.04	0.9982	0.9992	1

* MSRS is evaluated for all the algorithms except PM and C&C (log) which use MSRS_l_.

**Table 7:** Statistical comparison of biclusters identified in the yeast cell-cycle dataset using various algorithms

Algorithm	no. of biclusters	size*		no. of genes			no. of conditions		
				
		min	max	min	mean	max	min	mean	max
PA	25	10 × 5	597 × 17	10	97.64	597	5	13.16	17
PM	59	10 × 6	518 × 17	10	46.09	518	5	9.085	17
C&C	31	10 × 5	1391 × 17	10	91.74	1391	5	11.6	17
C&C (log)	5	12 × 13	2270 × 17	12	486.2	2270	13	14.80	17
ISA	18	28 × 5	149 × 6	28	74.56	149	5	5.667	7
OPSM	2	132 × 7	469 × 5	132	300.5	469	5	6	7
xMotifs	13	11 × 5	115 × 5	11	40.08	115	5	5	5

* The size of a bicluster is determined by its number of values, i.e. product of numbers of rows and columns.

When multiplicative model is concerned, i.e. the proposed algorithm for multiplicative model (PM) and C&C applied on log value (C&C (log)), the functional enrichment drops in general. At first glance, PM gives poorer performance in term of functional enrichment. Nonetheless, if the number of identified biclusters is also considered, PM actually outperforms C&C (log) by identifying more significant biclusters. The total number of biclusters identified by PM was 59 but CC only found 5 biclusters. In addition, the biclusters identified by PM exhibit higher homogeneity. The average values of MSRS_l _and ACV are 9.573 × 10^-3 ^and 0.9219 for PM respectively. In comparison, the average values of MSRS_l _and ACV are 6.262 × 10^-2 ^and 0.5740 for the C&C (log) respectively.

In addition to C&C based algorithms, Figure 14 shows the comparative results of ISA, OPSM and xMotifs for different values of *p*_0_. Although OPSM shows high percentage of functionally-enriched biclusters at large values of *p*_0_, there are only two biclusters found which are far from expectation. Thus, the proposed algorithms actually identify more functionally-enriched biclusters. Also, the percentage of functionally-enriched biclusters of OPSM drops to zero at low values of *p*_0_. At low values of *p*_0_, the results of ISA are the best in most cases. For *p*_0 _≥ 5 × 10^-4^, the performance of the proposed algorithm PA, however, is close to or even better than that of ISA. For both OPSM and ISA, the identified biclusters are less homogeneous in terms of average MSRS and ACV because their bicluster models are different from those studied in this paper. PA and PM show better performance than xMotifs in the percentage of functionally-enriched biclusters despite that our algorithms have lower average value of ACV. The reason is that xMotifs is designed to find biclusters with coherent state in each gene, which is only a subclass of additive models. The homogeneity analysis suggests that the difference in biological relevance of identified biclusters between various algorithms such as the proposed algorithm PA and ISA is not merely due to implementation architecture but also due to the model assumption.

In addition to the identification of biologically-significant biclusters, the efficiency of the proposed algorithm is justified by the processing time provided in Table 8. PA and PM require 0.72 and 1.35 sec respectively to finish. The results are the best and show improvement by a factor of at least 23.7 compared with the others. This implies that our algorithms have low computational complexity.

**Table 8:** Processing time for the yeast cell-cycle expression dataset using various biclustering algorithms

Algorithm	PA	PM	C&C	C&C (log)	ISA	OPSM	xMotifs
Processing time (sec)	0.72	1.35	32	1217	1590	38	937

Details of annotation results of the proposed algorithms PA and PM are shown in Tables 9, 10, 11 and Tables 12, 13, 14 at *p*-value < 0.001 respectively. In these tables, Bonferroni correction of *p*-value which adjusts the probability of random annotation for multiple tests [30] is provided. Consideration of the corrected *p*-value is important when multiple terms are tested for annotation in a single bicluster. The 4-th additive-related bicluster identified by PA has the lowest *p*-value in all the three ontologies. For the biological process ontology, 69 out of 201 genes are assigned to category "translation" at *p*-value of 6.92 × 10^-44^. The annotation is also significant after multiple test correction as it has a low corrected *p*-value of 8.51 × 10^-42^. For the cellular component ontology, 33 out of 201 genes are annotated with category "cytosolic small ribosomal subunit (sensu Eukaryota)" at *p*-value of 7.45 × 10^-29 ^(corrected *p*-value of 5.29 × 10^-27^). For the molecular function ontology, 66 out 201 genes are associated with category "structural constituent of ribosome" at *p*-value of 7.61 × 10^-47 ^(corrected *p*-value of 3.96 × 10^-45^). For the multiplicative model, the 24-th bicluster found by PM exhibits the lowest *p*-value in all the three ontologies. In fact, there are 141 genes shared between the biclusters with the lowest *p*-value identified by PA and PM, which correspond to 70.15% of genes in the bicluster identified by PA. As a result, the 24-th bicluster identified by PM are annotated with the similar categories as the 4-th bicluster found by PA in all the three ontologies. The annotations are also overrepresented in the bicluster as found in the experiments using PA except that the cellular component category with the lowest *p*-value is "cytosolic large ribosomal subunit (sensu Eukaryota)". The *p*-values are 6.16 × 10^-69^, 1.11 × 10^-45 ^and 3.69 × 10^-75 ^(corrected *p*-values of 7.51 × 10^-67^, 7.07 × 10^-44 ^and 1.88 × 10^-73^) while out of 225 genes there are 91, 46 and 88 genes annotated in the biological process, cellular component and molecular function categories respectively.

**Table 9:** Annotations of biological process ontology for biclusters identified by the proposed algorithm for additive models at p-value < 0.001.

Bicluster index	Annotation	P-value	Corrected P-value	Genes
1	chromatin modification	3.84E-04	1.39E-01	YBR081C, YBR198C, YDR392W, YDR448W, YGL112C, YMR236W, YNL097C
	histone acetylation	4.84E-04	1.76E-01	YBR081C, YBR198C, YDR392W, YDR448W, YFL039C, YGL112C, YJL081C, YMR236W, YNL136W
	endocytosis	7.24E-04	2.63E-01	YBR109C, YCL034W, YDR388W, YDR490C, YER166W, YFL039C, YGL106W, YHR001W, YHR073W, YNL084C, YNL227C, YNL243W, YOR089C, YOR109W, YOR327C, YPL145C
2	aerobic respiration	2.47E-04	4.34E-02	YBR026C, YDL174C, YDR231C, YHR001W, YMR030W, YMR081C, YPL132W, YPL159C
3	ribosome biogenesis and assembly	2.75E-13	4.73E-11	YBL024W, YBR142W, YDL153C, YDL167C, YDR060W, YDR120C, YDR312W, YDR365C, YEL026W, YER082C, YGL099W, YGR162W, YGR245C, YJL033W, YJR002W, YJR066W, YLL008W, YLR175W, YLR401C, YNL110C, YNL132W, YNL175C, YOL077C, YOR145C, YOR272W, YPL126W
	35S primary transcript processing	2.23E-06	3.84E-04	YCL031C, YDR339C, YER082C, YGR090W, YJL033W, YJR002W, YLL008W, YLR175W, YOR145C, YPR137W
4	ergosterol biosynthetic process	1.08E-04	1.33E-02	YLR450W, YML008C, YMR202W, YMR208W
	translational elongation	1.87E-06	2.30E-04	YAL003W, YDL081C, YDR382W, YDR385W, YLR249W, YLR340W, YOL039W
	regulation of translational fidelity	7.04E-07	8.67E-05	YBR048W, YDL229W, YDR025W, YGR118W, YNL209W, YPL081W
	ribosomal small subunit assembly and maintenance	6.90E-05	8.49E-03	YBR048W, YDR025W, YDR447C, YGR214W, YLR048W, YLR167W
	ribosomal large subunit assembly and maintenance	6.83E-06	8.40E-04	YBR142W, YDR312W, YLR075W, YLR340W, YLR448W, YML073C, YOL127W, YPR102C
	translation	6.92E-44	8.51E-42	YBL072C, YBL092W, YBR048W, YBR268W, YDL061C, YDL075W, YDL081C, YDL082W, YDL083C, YDL136W, YDL191W, YDL229W, YDR012W, YDR025W, YDR064W, YDR382W, YDR447C, YDR450W, YDR471W, YDR500C, YER074W, YER117W, YER131W, YGR118W, YGR214W, YHL001W, YHR141C, YIL069C, YJL136C, YJL190C, YJR123W, YKL056C, YKL156W, YKR057W, YKR094C, YLR048W, YLR075W, YLR167W, YLR185W, YLR325C, YLR340W, YLR388W, YLR441C, YLR448W, YML026C, YML063W, YML073C, YMR143W, YMR242C, YNL067W, YNL096C, YNL162W, YNL209W, YNL301C, YNL302C, YNL306W, YOL039W, YOL040C, YOL127W, YOR167C, YOR234C, YOR293W, YOR312C, YOR369C, YPL081W, YPL090C, YPL143W, YPL198W, YPR102C
6	protein folding	3.15E-04	1.51E-02	YDR214W, YFL016C, YML130C, YNL007C, YOR027W
	copper ion import	3.98E-04	1.91E-02	YLR411W, YPR124W
8	tricarboxylic acid cycle	8.65E-04	1.03E-01	YDR178W, YIL125W, YLL041C, YNL037C
	mitochondrial electron transport, ubiquinol to cytochrome c	4.23E-04	5.04E-02	YEL024W, YHR001W, YOR065W
	ubiquitin-dependent protein catabolic process	3.41E-04	4.05E-02	YBR173C, YDR394W, YJL001W, YMR119W, YOL038W
9	cytokinesis, contractile ring contraction	1.51E-04	3.17E-03	YBR038W, YHR023W
10	cell morphogenesis checkpoint	8.19E-04	7.37E-02	YJL187C, YKL101W
	chitin biosynthetic process	8.19E-04	7.37E-02	YER096W, YNL233W
	mitotic sister chromatid cohesion	6.67E-06	6.01E-04	YFL008W, YIL026C, YJL019W, YMR076C, YMR078C, YNL273W
14	glycolysis	4.18E-04	3.81E-02	YCL040W, YDR050C, YJR009C, YKL152C
	ribosomal small subunit assembly and maintenance	9.27E-04	8.43E-02	YDR337W, YGR214W, YLR167W, YML024W
15	protein folding	4.54E-04	1.22E-02	YAL005C, YBR169C, YDR214W, YLR216C
19	tricarboxylic acid cycle	7.49E-05	2.55E-03	YDR148C, YLL041C, YNR001C
24	response to stress	3.45E-04	3.45E-03	YBR072W, YDR258C, YPL240C
25	SRP-dependent cotranslational protein targeting to membrane, translocation	4.27E-04	8.12E-03	YAL005C, YER103W
	response to stress	6.85E-05	1.30E-03	YAL005C, YDR258C, YER103W, YPL240C
	protein folding	5.73E-04	1.09E-02	YAL005C, YDR258C, YER103W
	protein refolding	2.86E-04	5.43E-03	YAL005C, YPL240C

**Table 10:** Annotations of cellular component ontology for biclusters identified by the proposed algorithm for additive models at p-value < 0.001.

Bicluster index	Annotation	P-value	Corrected P-value	Genes
1	SLIK (SAGA-like) complex	4.45E-04	7.56E-02	YBR081C, YBR198C, YDR392W, YDR448W, YGL112C, YMR236W
	transcription factor TFIID complex	4.45E-04	7.56E-02	YBR198C, YER148W, YGL112C, YML114C, YMR236W, YPL129W
	INO80 complex	3.75E-04	6.38E-02	YDL002C, YFL039C, YJL081C, YLR052W, YPL129W
3	nucleolus	4.60E-14	4.28E-12	YAL059W, YBR142W, YCL031C, YCL054W, YDR312W, YDR339C, YDR365C, YDR378C, YEL026W, YEL055C, YGR090W, YGR159C, YJL033W, YJR002W, YLL008W, YLL034C, YLR175W, YML074C, YNL110C, YNL132W, YNL175C, YOL077C, YOL080C, YOR145C, YOR272W
	small nucleolar ribonucleoprotein complex	2.46E-04	2.29E-02	YDL153C, YDR378C, YEL026W, YER082C, YGR090W, YJR002W, YPL126W, YPR137W
4	cytosolic large ribosomal subunit (sensu Eukaryota)	1.76E-23	1.25E-21	YBL092W, YDL075W, YDL081C, YDL082W, YDL136W, YDL191W, YDR012W, YDR382W, YDR471W, YDR500C, YER117W, YHL001W, YHR141C, YKR094C, YLR075W, YLR185W, YLR325C, YLR340W, YLR448W, YML073C, YMR242C, YNL067W, YNL162W, YNL301C, YOL039W, YOL127W, YOR234C, YOR312C, YPL143W, YPL198W, YPR102C
	cytosolic small ribosomal subunit (sensu Eukaryota)	7.45E-29	5.29E-27	YBL072C, YBR048W, YDL061C, YDL083C, YDR025W, YDR064W, YDR447C, YDR450W, YER074W, YER131W, YGR118W, YGR214W, YIL069C, YJL136C, YJL190C, YJR123W, YKL156W, YKR057W, YLR048W, YLR167W, YLR388W, YLR441C, YML026C, YML063W, YMR143W, YNL096C, YNL302C, YOL040C, YOR167C, YOR293W, YOR369C, YPL081W, YPL090C
	ribosome	2.87E-05	2.04E-03	YAL003W, YDR385W, YEL034W, YKL056C, YLR249W, YOL139C, YPR163C
5	chromatin remodeling complex	5.68E-04	3.12E-02	YJL176C, YOR290C, YPL016W
8	mitochondrion	1.66E-08	1.11E-06	YBL015W, YBL090W, YBR003W, YBR037C, YBR120C, YBR122C, YBR147W, YCR028C, YDL027C, YDR141C, YDR178W, YDR305C, YDR316W, YDR494W, YDR513W, YEL006W, YEL024W, YER141W, YGL229C, YGR207C, YGR243W, YHR001W, YHR147C, YIL111W, YIL125W, YJL131C, YJL171C, YKL087C, YLL041C, YLR168C, YLR395C, YML120C, YMR145C, YMR167W, YNL037C, YNL073W, YOL038W, YOL059W, YOL096C, YOR065W, YOR317W, YOR356W, YOR386W, YPL005W, YPL029W, YPL103C
	respiratory chain complex III (sensu Eukaryota)	4.23E-04	2.84E-02	YEL024W, YHR001W, YOR065W
	endosome	9.02E-05	6.04E-03	YAL030W, YDL113C, YJL053W, YLR119W, YLR408C, YNR006W, YOR036W
9	bud neck	1.42E-06	2.27E-05	YBR038W, YGR092W, YHR023W, YIL106W, YLR190W, YOL070C
10	bud neck	7.51E-04	3.68E-02	YDR507C, YGR152C, YGR238C, YIL140W, YJL187C, YKL101W, YNL233W
	septin ring	9.01E-05	4.42E-03	YIL140W, YKL101W, YNL233W
14	lipid particle	5.85E-05	2.93E-03	YIL124W, YJR009C, YMR110C, YNL231C, YOR317W
17	mitochondrion	8.16E-06	2.86E-04	YCL057W, YDL027C, YDL164C, YDR116C, YDR194C, YDR375C, YDR513W, YGL104C, YHR002W, YHR067W, YHR147C, YIL087C, YIL111W, YJL063C, YLL040C, YLR270W, YLR346C, YMR098C, YMR152W, YMR188C, YNL063W, YNL073W, YNL200C, YNL274C, YOL059W, YOL071W, YOR136W, YPR011C
18	bud neck contractile ring	6.08E-05	1.58E-03	YHR023W, YJR092W, YMR032W
	pre-autophagosomal structure	6.62E-04	1.72E-02	YBL078C, YDL113C
20	nuclear cohesin complex	3.74E-04	5.24E-03	YDL003W, YIL026C

**Table 11:** Annotations of molecular function ontology for biclusters identified by the proposed algorithm for additive models at p-value < 0.001.

Bicluster index	Annotation	P-value	Corrected P-value	Genes
1	endopeptidase activity	7.71E-05	1.26E-02	YBL041W, YDR394W, YER012W, YJL001W, YOL038W, YOR362C
3	ATP-dependent RNA helicase activity	5.10E-04	3.57E-02	YBR142W, YER172C, YJL033W, YLL008W, YMR080C
	snoRNA binding	7.57E-04	5.30E-02	YDL153C, YER082C, YGR090W, YPL126W, YPR137W
4	structural constituent of ribosome	7.61E-47	3.96E-45	YBL072C, YBL092W, YBR048W, YBR268W, YDL061C, YDL075W, YDL081C, YDL082W, YDL083C, YDL136W, YDL191W, YDR012W, YDR025W, YDR064W, YDR382W, YDR447C, YDR450W, YDR471W, YDR500C, YER074W, YER117W, YER131W, YGR118W, YGR214W, YHL001W, YHR141C, YIL069C, YJL136C, YJL190C, YJR123W, YKL156W, YKR057W, YKR094C, YLR048W, YLR075W, YLR167W, YLR185W, YLR325C, YLR340W, YLR388W, YLR441C, YLR448W, YML026C, YML063W, YML073C, YMR143W, YMR242C, YNL067W, YNL096C, YNL162W, YNL301C, YNL302C, YNL306W, YOL039W, YOL040C, YOL127W, YOR167C, YOR234C, YOR293W, YOR312C, YOR369C, YPL081W, YPL090C, YPL143W, YPL198W, YPR102C
	RNA-directed DNA polymerase activity	9.72E-04	5.05E-02	YAR009C, YJR027W, YML039W, YML045W, YMR045C, YMR050C
	DNA helicase activity	5.11E-04	2.66E-02	YDR545W, YLR467W, YNL339C, YPL283C, YPR204W
	ribonuclease activity	9.72E-04	5.05E-02	YAR009C, YJR027W, YML039W, YML045W, YMR045C, YMR050C
	RNA binding	3.10E-05	1.61E-03	YAR009C, YDL208W, YDR378C, YDR381W, YEL026W, YHL001W, YJR027W, YLR277C, YLR448W, YML039W, YML045W, YML073C, YMR045C, YMR050C, YNL175C, YOL123W, YOL127W
	helicase activity	2.93E-05	1.53E-03	YEL077C, YJL225C, YLL066C, YLL067C, YML133C
6	copper uptake transporter activity	3.98E-04	1.11E-02	YLR411W, YPR124W
17	glycerol-3-phosphate dehydrogenase (NAD+) activity	8.19E-04	2.95E-02	YDL022W, YOL059W
18	spermidine transporter activity	1.12E-04	2.24E-03	YLL028W, YOR273C
	spermine transporter activity	6.62E-04	1.32E-02	YLL028W, YOR273C
24	unfolded protein binding	1.05E-04	4.19E-04	YBR072W, YDR258C, YPL240C
25	unfolded protein binding	1.38E-05	8.29E-05	YAL005C, YDR258C, YER103W, YPL240C
	ATPase activity	9.71E-04	5.82E-03	YAL005C, YDR258C, YER103W

**Table 12:** Annotations of biological process ontology for biclusters identified by the proposed algorithm for multiplicative models at p-value < 0.001.

Bicluster index	Annotation	P-value	Corrected P-value	Genes
1	ribosomal large subunit biogenesis and assembly	5.83E-06	1.97E-03	YAL025C, YBR267W, YDR091C, YNL110C, YNL163C, YOR272W, YPL211W
	tRNA methylation	1.94E-04	6.55E-02	YBL024W, YBR061C, YDR165W, YNR046W, YOL093W, YOL124C
	processing of 20S pre-rRNA	1.80E-05	6.05E-03	YDL153C, YDL166C, YDR449C, YEL026W, YJL191W, YJR002W, YLR068W, YLR192C, YLR222C, YML093W, YMR093W, YOR056C, YPR137W
	transcription from RNA polymerase III promoter	1.02E-04	3.45E-02	YBR154C, YDL150W, YDR045C, YER148W, YLR223C, YNL113W, YNR003C, YOR224C
	35S primary transcript processing	6.37E-06	2.15E-03	YBL004W, YCL031C, YCL059C, YDR339C, YGR090W, YJR002W, YKR060W, YLL008W, YLR051C, YLR186W, YLR430W, YNR038W, YOL021C, YOR145C, YPR112C, YPR137W
	transcription from RNA polymerase I promoter	9.82E-04	3.31E-01	YBL014C, YBR154C, YDR156W, YER148W, YNL113W, YOR224C, YOR341W
	rRNA processing	5.33E-04	1.80E-01	YBR142W, YBR257W, YCL059C, YDR365C, YDR478W, YGR159C, YLR223C, YMR049C, YMR290C, YOL144W, YOR145C, YPL211W
	ribosome biogenesis and assembly	1.09E-15	3.66E-13	YAL025C, YBL024W, YBL054W, YBR034C, YBR084W, YBR142W, YBR267W, YCL059C, YDL153C, YDL167C, YDR060W, YDR165W, YDR300C, YDR312W, YDR365C, YDR449C, YDR465C, YEL026W, YHL039W, YJR002W, YJR066W, YKL143W, YKL191W, YKR056W, YKR060W, YLL008W, YLR186W, YML093W, YMR093W, YMR131C, YMR290C, YNL110C, YNL113W, YNL132W, YNL175C, YNR003C, YNR038W, YNR053C, YOL077C, YOL124C, YOL144W, YOR056C, YOR145C, YOR206W, YOR272W, YPL211W, YPL212C, YPL226W
	mRNA export from nucleus	3.51E-04	1.18E-01	YBR034C, YDL116W, YDR432W, YER107C, YJL140W, YKL057C, YKL068W, YKR002W, YKR095W, YMR308C, YOR098C
5	nucleotide-excision repair	1.88E-04	1.97E-02	YBR088C, YDL164C, YJL173C, YNL312W
	DNA recombination	1.47E-05	1.54E-03	YDL164C, YJL173C, YML061C, YNL312W
	DNA replication, synthesis of RNA primer	6.91E-05	7.25E-03	YJL173C, YKL045W, YNL312W
	double-strand break repair via homologous recombination	8.96E-04	9.41E-02	YER147C, YJL173C, YNL312W
7	arabinose catabolic process	3.55E-04	1.03E-02	YHR104W, YOR120W
	D-xylose catabolic process	3.55E-04	1.03E-02	YHR104W, YOR120W
	protein refolding	1.22E-05	3.55E-04	YBR169C, YLL026W, YPL240C
15	protein refolding	2.41E-06	6.74E-05	YAL005C, YBR169C, YPL240C
21	ribosome biogenesis and assembly	2.26E-04	1.26E-02	YAL025C, YBR238C, YBR267W, YDL031W, YDR083W, YDR184C, YIR026C, YKL078W
24	Glycolysis	3.40E-05	4.15E-03	YAL038W, YCR012W, YDR050C, YJR009C, YKL060C, YKL152C
	translational elongation	4.41E-09	5.38E-07	YAL003W, YBR118W, YDL081C, YDL130W, YDR382W, YDR385W, YLR249W, YLR340W, YOL039W
	regulation of translational fidelity	1.61E-08	1.97E-06	YBR048W, YBR189W, YDL229W, YDR025W, YGR118W, YNL209W, YPL081W
	ribosomal small subunit assembly and maintenance	4.58E-07	5.59E-05	YBR048W, YCR031C, YDR025W, YDR447C, YGR214W, YLR048W, YLR167W, YML024W
	ribosomal large subunit assembly and maintenance	1.58E-04	1.93E-02	YDR418W, YLR075W, YLR340W, YLR448W, YML073C, YOL127W, YPR102C
	translation	6.16E-69	7.51E-67	YBL027W, YBL038W, YBL072C, YBL092W, YBR031W, YBR048W, YBR181C, YBR189W, YBR191W, YCR031C, YDL061C, YDL075W, YDL081C, YDL082W, YDL083C, YDL130W, YDL136W, YDL191W, YDL229W, YDR012W, YDR025W, YDR064W, YDR382W, YDR418W, YDR447C, YDR450W, YDR471W, YDR500C, YER074W, YER102W, YER117W, YER131W, YGR118W, YGR214W, YHR141C, YIL069C, YJL136C, YJL177W, YJL189W, YJL190C, YJR123W, YJR145C, YKL006W, YKL056C, YKL156W, YKL180W, YKR057W, YKR094C, YLL045C, YLR029C, YLR048W, YLR075W, YLR167W, YLR185W, YLR325C, YLR333C, YLR340W, YLR344W, YLR388W, YLR406C, YLR441C, YLR448W, YML024W, YML026C, YML063W, YML073C, YMR121C, YMR143W, YMR194W, YMR230W, YMR242C, YNL067W, YNL096C, YNL162W, YNL209W, YNL301C, YNL302C, YOL039W, YOL040C, YOL127W, YOR167C, YOR234C, YOR293W, YOR312C, YOR369C, YPL081W, YPL090C, YPL143W, YPL198W, YPR043W, YPR102C
	telomere maintenance via recombination	5.04E-04	6.14E-02	YDR545W, YER190W, YLR467W, YNL339C, YPL283C
31	nucleotide-excision repair	3.95E-04	2.05E-02	YAR007C, YDL164C, YNL312W
	DNA recombination	6.57E-05	3.42E-03	YAR007C, YDL164C, YNL312W
	DNA replication, synthesis of RNA primer	9.46E-04	4.92E-02	YAR007C, YNL312W
	mitotic sister chromatid cohesion	6.18E-05	3.21E-03	YDL003W, YFL008W, YIL026C, YMR078C
	DNA strand elongation during DNA replication	7.82E-07	4.06E-05	YAR007C, YKL108W, YLR103C, YNL312W
34	NADH oxidation	1.25E-05	9.00E-04	YBR145W, YML120C, YMR145C, YOL059W
35	transposition, RNA-mediated	3.41E-06	1.36E-04	YCL020W, YER160C, YJR026W, YJR028W, YML040W, YOR142W
36	glycine catabolic process	1.94E-05	9.31E-04	YAL044C, YDR019C, YMR189W
	one-carbon compound metabolic process	4.79E-05	2.30E-03	YAL044C, YDR019C, YMR189W
41	karyogamy during conjugation with cellular fusion	4.10E-04	3.86E-02	YCL055W, YNL313C, YPL192C
	ribosome biogenesis and assembly	2.32E-05	2.18E-03	YBR267W, YCR072C, YDL031W, YDR184C, YDR465C, YGL099W, YGR187C, YMR128W, YOL010W, YOR001W
	35S primary transcript processing	4.81E-04	4.52E-02	YDL031W, YGR090W, YOL010W, YOL021C, YOR001W
50	pseudohyphal growth	9.72E-04	4.57E-02	YBR083W, YJL164C, YKL185W, YOR127W
	N-terminal protein myristoylation	5.58E-04	2.62E-02	YIL009W, YOR317W
54	DNA unwinding during replication	6.79E-04	4.21E-02	YBR202W, YGL201C, YLR274W
	DNA replication initiation	1.69E-04	1.05E-02	YBL035C, YBR202W, YGL201C, YLR274W
	pheromone-dependent signal transduction during conjugation with cellular fusion	2.18E-04	1.35E-02	YHR005C, YJL157C, YNL173C, YOR127W
55	spore wall assembly (sensu Fungi)	7.33E-05	3.66E-03	YDR126W, YDR523C, YOR177C, YOR242C
56	meiotic mismatch repair	3.03E-05	1.48E-03	YDR097C, YNL082W, YOL090W
	mismatch repair	6.19E-04	3.03E-02	YDR097C, YNL082W, YOL090W
	microtubule nucleation	1.13E-04	5.52E-03	YDR356W, YKL042W, YOR373W, YPL124W
57	sulfate assimilation	1.76E-04	9.50E-03	YFR030W, YJR010W, YKR069W
	microtubule nucleation	1.24E-04	6.67E-03	YBL063W, YMR117C, YOR373W, YPL124W
59	DNA replication checkpoint	5.86E-04	3.23E-02	YCL061C, YMR048W

**Table 13:** Annotations of cellular component ontology for biclusters identified by the proposed algorithm for multiplicative models at p-value < 0.001.

Bicluster index	Annotation	P-value	Corrected P-value	Genes
1	DNA-directed RNA polymerase I complex	9.40E-04	1.50E-01	YBR154C, YDR156W, YNL113W, YOR224C, YOR341W
	nucleoplasm	6.60E-04	1.06E-01	YAL059W, YDL051W, YKR002W, YKR095W, YNL175C, YNR053C
	nucleolus	9.69E-17	1.55E-14	YAL025C, YAL059W, YBL004W, YBL026W, YBR142W, YCL031C, YCL054W, YCL059C, YDL051W, YDR299W, YDR312W, YDR339C, YDR365C, YDR378C, YEL026W, YGR090W, YGR159C, YJR002W, YKR060W, YLL008W, YLL034C, YLR051C, YLR068W, YLR186W, YLR223C, YMR049C, YMR131C, YMR233W, YMR269W, YMR290C, YNL110C, YNL132W, YNL147W, YNL175C, YNL299W, YNR038W, YNR046W, YNR053C, YOL041C, YOL077C, YOL144W, YOR145C, YOR272W, YPL211W, YPR112C
	nucleus	1.60E-05	2.56E-03	YAL059W, YAR015W, YBL016W, YBL024W, YBL054W, YBL093C, YBR034C, YBR066C, YBR090C, YBR112C, YBR160W, YBR173C, YCL011C, YCL031C, YCL054W, YCR036W, YCR051W, YCR059C, YCR060W, YCR090C, YDL002C, YDL006W, YDL047W, YDL051W, YDL070W, YDL076C, YDL153C, YDL166C, YDR006C, YDR091C, YDR098C, YDR143C, YDR155C, YDR162C, YDR165W, YDR260C, YDR296W, YDR305C, YDR361C, YDR365C, YDR390C, YDR432W, YDR465C, YDR477W, YEL007W, YER012W, YER042W, YER148W, YGL130W, YGR090W, YGR159C, YGR200C, YJL140W, YJR002W, YJR017C, YJR105W, YKL143W, YKR060W, YKR072C, YKR079C, YKR096W, YLL034C, YLR007W, YLR039C, YLR051C, YLR052W, YLR068W, YLR107W, YLR186W, YLR223C, YLR262C, YLR265C, YLR327C, YLR384C, YLR420W, YLR430W, YML032C, YML053C, YML080W, YML081W, YML114C, YMR009W, YMR021C, YMR049C, YMR070W, YMR074C, YMR092C, YMR176W, YMR178W, YMR226C, YMR233W, YMR235C, YMR308C, YNL004W, YNL016W, YNL110C, YNL136W, YNL164C, YNL186W, YNL199C, YNL215W, YNL299W, YNR003C, YNR046W, YNR053C, YOL093W, YOL108C, YOL143C, YOR006C, YOR056C, YOR123C, YOR145C, YOR189W, YOR206W, YOR252W, YOR272W, YOR283W, YOR304W, YPL047W, YPL086C, YPL204W, YPL212C, YPL268W, YPR069C, YPR073C
	small nucleolar ribonucleoprotein complex	1.97E-05	3.16E-03	YBL004W, YBL026W, YCL059C, YDL153C, YDR378C, YDR449C, YEL026W, YGR090W, YJL191W, YJR002W, YLR186W, YLR222C, YML093W, YMR093W, YNL147W, YPR137W
5	incipient bud site	4.75E-04	2.18E-02	YGR189C, YKR090W, YLL021W, YNL233W, YNL304W
	nucleus	1.26E-05	5.80E-04	YBL046W, YBR073W, YBR088C, YCR065W, YDL006W, YDL103C, YDL164C, YDL197C, YER003C, YER152C, YGR042W, YKL045W, YKL089W, YKL113C, YLL022C, YLR233C, YLR376C, YML021C, YML061C, YML109W, YOR074C, YOR279C, YOR342C, YPL008W, YPL127C, YPL208W, YPL256C, YPR120C, YPR135W
	bud neck	4.38E-04	2.01E-02	YDR507C, YGR152C, YKL101W, YKR090W, YLL021W, YNL233W, YNL304W
11	MCM complex	8.91E-04	5.52E-02	YEL032W, YLR274W, YPR019W
12	bud neck	1.07E-05	3.33E-04	YBR038W, YBR200W, YGR092W, YHR023W, YIL106W, YLR190W, YMR001C, YPR119W
14	spindle microtubule	6.05E-05	2.12E-03	YBL063W, YBR156C, YGL061C
16	endoplasmic reticulum	1.91E-04	4.21E-03	YBR229C, YCR011C, YCR044C, YDL204W, YIL124W, YML128C, YMR134W
	mitochondrion	7.89E-04	1.74E-02	YBR003W, YBR026C, YBR037C, YBR147W, YBR229C, YCR005C, YHL021C, YHR067W, YIL087C, YIL124W, YLR142W, YLR253W, YML128C, YNL073W
24	cytosolic large ribosomal subunit (sensu Eukaryota)	1.11E-45	7.07E-44	YBL027W, YBL092W, YBR031W, YBR191W, YDL075W, YDL081C, YDL082W, YDL130W, YDL136W, YDL191W, YDR012W, YDR382W, YDR418W, YDR471W, YDR500C, YER117W, YHR141C, YJL177W, YJL189W, YKL006W, YKL180W, YKR094C, YLL045C, YLR029C, YLR075W, YLR185W, YLR325C, YLR340W, YLR344W, YLR406C, YLR448W, YML073C, YMR121C, YMR194W, YMR242C, YNL067W, YNL162W, YNL301C, YOL039W, YOL127W, YOR234C, YOR312C, YPL143W, YPL198W, YPR043W, YPR102C
	cytosolic small ribosomal subunit (sensu Eukaryota)	7.45E-43	4.77E-41	YBL072C, YBR048W, YBR181C, YBR189W, YCR031C, YDL061C, YDL083C, YDR025W, YDR064W, YDR447C, YDR450W, YER074W, YER102W, YER131W, YGR118W, YGR214W, YIL069C, YJL136C, YJL190C, YJR123W, YJR145C, YKL156W, YKR057W, YLR048W, YLR167W, YLR333C, YLR388W, YLR441C, YML024W, YML026C, YML063W, YMR116C, YMR143W, YMR230W, YNL096C, YNL302C, YOL040C, YOR167C, YOR293W, YOR369C, YPL081W, YPL090C
	ribosome	4.84E-06	3.10E-04	YAL003W, YBR118W, YDR385W, YEL034W, YKL056C, YLR249W, YOL139C, YPR163C
28	condensed nuclear chromosome	2.75E-04	2.20E-03	YHR157W, YPL194W
31	chromosome, telomeric region	4.77E-04	1.43E-02	YAR007C, YNL312W
	nuclear cohesin complex	3.79E-05	1.14E-03	YDL003W, YFL008W, YIL026C
	DNA replication factor A complex	4.77E-04	1.43E-02	YAR007C, YNL312W
	replication fork	2.99E-04	8.97E-03	YDL164C, YKL108W, YLR103C
34	mitochondrial inner membrane	4.76E-04	2.14E-02	YDL198C, YDR197W, YER058W, YER141W, YOL027C, YPR011C
	mitochondrion	5.60E-06	2.52E-04	YDL198C, YDR194C, YDR197W, YDR301W, YDR322W, YDR505C, YER058W, YER141W, YGL187C, YHR147C, YJR048W, YKL150W, YLR168C, YML030W, YML052W, YML120C, YMR098C, YMR145C, YMR188C, YNL306W, YNR036C, YOL009C, YOL027C, YOL038W, YOL059W, YPR011C
35	retrotransposon nucleocapsid	3.41E-06	9.89E-05	YCL020W, YER160C, YJR026W, YJR028W, YML040W, YOR142W
36	glycine cleavage complex	1.94E-05	4.46E-04	YAL044C, YDR019C, YMR189W
41	nuclear exosome (RNase complex)	4.93E-05	2.02E-03	YNL251C, YOL021C, YOR001W
54	MCM complex	1.19E-04	4.65E-03	YBR202W, YGL201C, YLR274W
	pre-replicative complex	9.21E-04	3.59E-02	YBR202W, YGL201C, YLR274W
56	central plaque of spindle pole body	3.03E-05	8.78E-04	YDR356W, YKL042W, YPL124W

**Table 14:** Annotations of molecular function ontology for biclusters identified by the proposed algorithm for multiplicative models at p-value < 0.001.

Bicluster index	Annotation	P-value	Corrected P-value	Genes
1	DNA-directed RNA polymerase activity	1.62E-05	2.62E-03	YBR154C, YDL140C, YDL150W, YDR045C, YDR156W, YJL140W, YNL113W, YNR003C, YOR224C, YOR341W
	snoRNA binding	1.55E-04	2.51E-02	YBL004W, YDL153C, YDR449C, YGR090W, YLR222C, YML093W, YMR093W, YPR112C, YPR137W
3	MAP kinase kinase activity	9.00E-04	4.50E-02	YJL128C, YPL140C
7	ATPase activity, coupled	3.55E-04	6.04E-03	YLL026W, YPL240C
	aldo-keto reductase activity	3.55E-04	6.04E-03	YHR104W, YOR120W
11	chromatin binding	6.21E-05	3.66E-03	YEL032W, YJL081C, YLR002C, YLR274W, YPR019W
14	protein phosphatase type 2C activity	4.51E-04	6.77E-03	YCR079W, YDL006W
24	structural constituent of ribosome	3.69E-75	1.88E-73	YBL027W, YBL038W, YBL072C, YBL092W, YBR031W, YBR048W, YBR181C, YBR189W, YBR191W, YCR031C, YDL061C, YDL075W, YDL081C, YDL082W, YDL083C, YDL130W, YDL136W, YDL191W, YDR012W, YDR025W, YDR064W, YDR382W, YDR418W, YDR447C, YDR450W, YDR471W, YDR500C, YER074W, YER102W, YER117W, YER131W, YGR118W, YGR214W, YHR141C, YIL069C, YJL136C, YJL177W, YJL189W, YJL190C, YJR123W, YJR145C, YKL006W, YKL156W, YKL180W, YKR057W, YKR094C, YLL045C, YLR029C, YLR048W, YLR075W, YLR167W, YLR185W, YLR325C, YLR333C, YLR340W, YLR344W, YLR388W, YLR406C, YLR441C, YLR448W, YML024W, YML026C, YML063W, YML073C, YMR121C, YMR143W, YMR194W, YMR230W, YMR242C, YNL067W, YNL096C, YNL162W, YNL301C, YNL302C, YOL039W, YOL040C, YOL127W, YOR167C, YOR234C, YOR293W, YOR312C, YOR369C, YPL081W, YPL090C, YPL143W, YPL198W, YPR043W, YPR102C
	translation elongation factor activity	4.78E-04	2.44E-02	YAL003W, YBR118W, YDR385W, YLR249W
	DNA helicase activity	6.99E-05	3.57E-03	YDR545W, YER190W, YLR467W, YNL339C, YPL283C, YPR204W
	RNA binding	4.35E-04	2.22E-02	YAR009C, YCR031C, YDL208W, YJR027W, YKL006W, YLR029C, YLR344W, YLR448W, YML039W, YML045W, YML073C, YMR045C, YMR050C, YMR121C, YMR194W, YOL127W
	helicase activity	1.61E-08	8.22E-07	YBL113C, YEL077C, YIL177C, YJL225C, YLL066C, YLL067C, YML133C
35	RNA binding	3.28E-05	1.05E-03	YCL020W, YER160C, YJR026W, YJR028W, YML040W, YMR290C, YOR142W, YPR107C
36	glycine dehydrogenase (decarboxylating) activity	1.94E-05	5.62E-04	YAL044C, YDR019C, YMR189W
50	long-chain-fatty-acid-CoA ligase activity	5.58E-04	1.06E-02	YIL009W, YOR317W
	glycerol-3-phosphate dehydrogenase (NAD+) activity	1.88E-04	3.56E-03	YDL022W, YOL059W
	citrate (Si)-synthase activity	5.58E-04	1.06E-02	YCR005C, YNR001C
52	structural constituent of cytoskeleton	5.63E-04	9.57E-03	YDR016C, YGR113W, YHR129C, YNL126W
54	ATP-dependent DNA helicase activity	3.25E-04	1.11E-02	YBR202W, YGL201C, YLR274W
	ATP binding	3.25E-04	1.11E-02	YBR202W, YDR097C, YNL082W
56	ATP binding	1.64E-04	3.78E-03	YDR097C, YNL082W, YOL090W
	structural constituent of cytoskeleton	4.43E-05	1.02E-03	YDR356W, YGR113W, YKL042W, YOR373W, YPL124W
	guanine/thymine mispair binding	2.17E-04	5.00E-03	YDR097C, YOL090W
	single base insertion or deletion binding	2.17E-04	5.00E-03	YDR097C, YOL090W
	four-way junction DNA binding	2.17E-04	5.00E-03	YDR097C, YOL090W
57	structural constituent of cytoskeleton	7.39E-04	1.85E-02	YBL063W, YMR117C, YOR373W, YPL124W
58	copper ion binding	6.45E-04	1.42E-02	YBR037C, YBR295W
59	guanine/thymine mispair binding	1.97E-04	4.73E-03	YDR097C, YOL090W
	four-way junction DNA binding	1.97E-04	4.73E-03	YDR097C, YOL090W
	single base insertion or deletion binding	1.97E-04	4.73E-03	YDR097C, YOL090W

The experiments on the real dataset show that our proposed algorithms PA and PM can identify biclusters with high biological relevance efficiently. Furthermore, PA can always give a reasonable number of biclusters, and with a good degree of homogeneity. Although GO annotation only provides descriptions currently known in the biological community, the results still give a reasonable indication of performance. Furthermore, the biclusters which have no GO terms assigned should be investigated for any new biological discoveries.

### Determination of biclusters homogeneity

**Figure 15:**
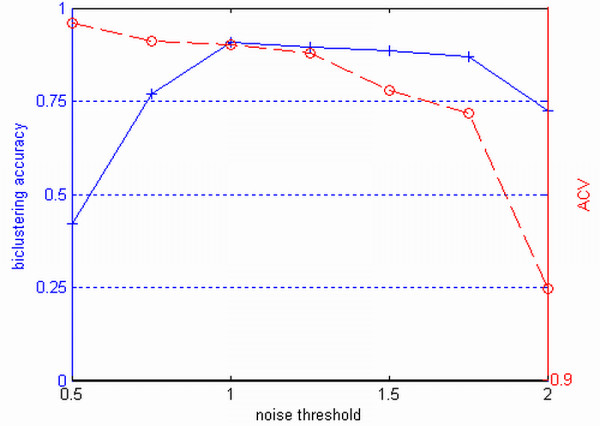
Biclustering accuracy (solid line) and the ACV (dashed line) against noise threshold *ε*.

In previous experiments, the homogeneity parameter, i.e. noise threshold *ε *of our algorithms is determined empirically. In fact, the aforementioned exploratory approach based on the PC plots can be employed to determine this parameter in an interactive manner for a given dataset. This exploratory approach uses an assumption that the homogeneity decreases monotonically with *ε *while the biclustering accuracy is a concave function of *ε*. To see this, we apply the proposed algorithm for additive models to artificial datasets with noise s.d. of 0.3 over a wide range of *ε*. Figure 15 shows the graphs of biclustering accuracy and ACV against *ε*. The biclustering accuracy first rises rapidly to its maximum value when *ε *changes from 0.5 to 1. The biclustering accuracy then decreases slightly until *ε *becomes 1.75. A steeper drop is found when *ε *is larger than 1.75. In other words, the biclustering accuracy is approximately concave with respect to *ε*. On the other hand, when *ε *increases, the average ACV of detected biclusters decreases as expected. From the graph, it can be observed that the ACV decreases faster when *ε *exceeds 1.25. Meanwhile, the biclustering accuracy remains high for *ε *between 1 and 1.25. These observations support the use of the interactive approach for parameter determination.

## Conclusion

In this paper, a novel biclustering algorithm for additive models is proposed. First, we performed analysis on the difference matrix computed from a gene expression matrix. It was shown that the column-wise differences of an additive-related bicluster appear as clusters in each corresponding column in the difference matrix. Similarly, clusters can be found from the column-wise ratios calculated from multiplicative-related biclusters. These observations were then explored to construct biclusters greedily from the clustering results in column-wise differences or ratios in the proposed algorithms.

The proposed algorithms have been analyzed by comparing with pCluster algorithm. The results suggest that the proposed algorithms can be regarded as a greedy version of the pCluster algorithm. The biclusters found by the proposed algorithms can be expressed as *δ*-pClusters but clustering density is utilized in pattern discovery. Although the identified *δ*-pClusters is not guaranteed to be maximal, the proposed algorithm is much more efficient. Experiments showed that the computational time of the proposed algorithms is lower than that of the pCluster algorithm by a factor of hundreds or more. Moreover, we have verified that the worst case complexity of the proposed algorithms is polynomial-time instead of exponential-time as in the case of the pCluster algorithm or other *δ*-pCluster based approaches.

The robustness of our algorithms to noise and regulatory complexity has been verified empirically using artificial datasets. It was found that our algorithm is capable of discovering overlapping biclusters under noisy condition. Biological significance of biclustering results has been verified on the yeast cell-cycle dataset using Gene Ontology annotations. Comparative study shows that the proposed algorithm is the best or close to be the best one among several existing algorithms in terms of the percentage and the number of functionally-enriched biclusters for *p*-values below a range of value from 5 × 10^-3 ^to 5 × 10^-2^. In particular, there are 96.0%, 88.0% and 80.0% of the biclusters annotated with *p*-value below 0.01. The proposed algorithm can identify biclusters with less deviation from the additive models. The identified biclusters also have reasonable size ranged from 10 to 597 genes and 5 to 17 conditions. Comparison in processing time suggests that the proposed algorithm has the highest efficiency.

In the proposed algorithm, the noise threshold is a crucial parameter as it balances the homogeneity requirement and the noise tolerance in the identified biclusters. In order to determine an appropriate value for the noise threshold, an exploratory approach based on the PC plots is adopted. We believe that the proposed biclustering algorithm and the interactive PC plots offer an effective data analysis tool for gene expression data. In future, our research will be focused on detecting bicluster types other than additive or multiplicative models, e.g. biclusters of coherent evolution.

## Availability and requirements

Project home page: .

Operating system: Window XP

Programming language: Matlab 6.5 or above

License: Free for academic use. For non-academic use, please contact the author.

## Authors' contributions

KOC worked on the proposed biclustering algorithm, implementation and experimental analysis. NFL formulated the biclustering problem and designed the algorithm. WCS initiated the project. AWCL worked on the experimental analysis. All authors read and approved the final manuscript.

## Supplementary Material

Additional File 1 **Supplementary materials**. This file contains the content about the parallel coordinate (PC) representation of biclusters given in Figure 1 in the article, which can serve as preliminary for readers who are unfamiliar with PC plots. It also discusses visualization of biclusters in a data matrix which may help reader to understand the interactive adjustment of noise threshold for the proposed algorithm described in the article. Click here for file
