# Supplementary material for: Identification of coherent patterns in gene expression data using an efficient biclustering algorithm and parallel coordinate visualization
Source: BMC Bioinformatics. 2008 Apr 23;9:210. doi: 10.1186/1471-2105-9-210 (PMC2396181; doi:10.1186/1471-2105-9-210)
Supplement: Additional File 1 — Supplementary materials. This file contains the content about the parallel coordinate (PC) representation of biclusters given in Figure 1 in the article, which can serve as preliminary for readers who are unfamiliar with PC plots. It also discusses visualization of biclusters in a data matrix which may help reader to understand the interactive adjustment of noise threshold for the proposed algorithm described in the article. [file 1471-2105-9-210-S1.pdf]

Supplementary material

Identification of Coherent Patterns in Gene Expression Data Using an Efficient Biclustering Algorithm and Parallel Coordinate Visualization

Kin-On Cheng[1], Ngai-Fong Law[1], Wan-Chi Siu[1], Alan Wee-Chung Liew[2§]

[1]Centre for Signal Processing, Department of Electronic and Information Engineering, The Hong Kong Polytechnic University, Hung Hom, Hong Kong
[2]School of Information and Communication Technology, Griffith University, Gold Coast Campus, QLD 4222, Queensland, Australia

[§]Corresponding author

Biclusters representation in parallel coordinate plots

The parallel coordinate (PC) plots of patterns shown in Figure 1 are given in Figure 2. Although the numerical values of these patterns appear to be substantially different from each other, the patterns exhibit certain structures in the PC plots. The constant bicluster shows an overlapped line across all the columns where the number of overlapped lines is determined by the number of rows. The constant row bicluster shows zero slope lines across all the columns where the number of lines is again determined by the number of rows. The constant column bicluster shows an overlapped non-zero slope line across all the columns. The additive-related bicluster shows a number of non-zero slope lines across all the columns. Note that the slopes for these lines are the same in the PC plot as can be seen in Figure 2 (D). Figure 2 (E) shows the PC plot for the multiplicative-related bicluster. At first glance, no special structure can be observed. However, if {C2/C1, C3/C1} is used for the PC plot as in Figure 2 (F), we can see that an overlapped line is obtained. Therefore, these different types of biclusters exhibit similar structures as depicted in the PC plot. These special structures allow us to distinguish biclusters from unrelated expression values in the PC plot. Also, from these structures, we can predict the type of biclusters present.

(A)

C1	C2	C3
5	5	5
5	5	5
5	5	5

(B)

C1	C2	C3
25	25	25
7	7	7
16	16	16

(C)

C1	C2	C3
25	7	16
25	7	16
25	7	16

(D)

C1	C2	C3
2	4	5
3	5	6
4	6	7

(E)

C1	C2	C3
45	9	18
5	1	2
40	8	16

Figure 1 - Examples of different biclusters. (A) A constant bicluster. (B) A constant row bicluster. (C) A constant column bicluster. (D) An additive-related bicluster. (E) A multiplicative-related bicluster. Note that *Ci* denotes the *i*-th experimental condition.

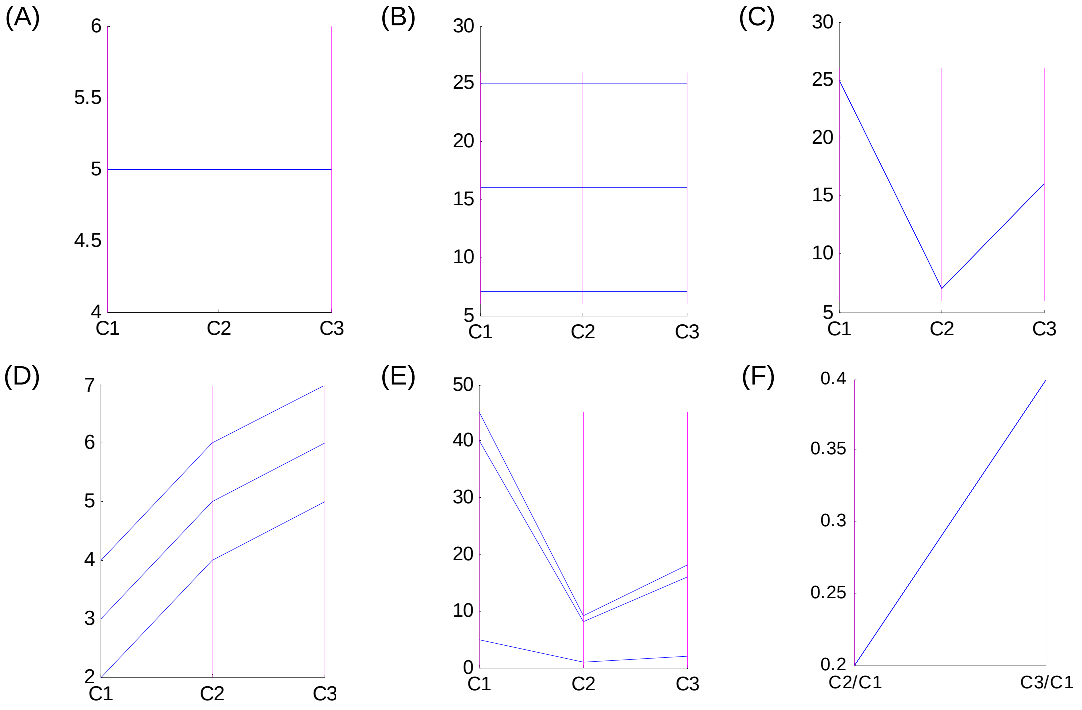

Figure 2 – Parallel coordinate (PC) plots for biclusters in Figure 1. (A) A constant bicluster. (B) A constant row bicluster. (C) A constant column bicluster. (D) An additive-related bicluster. (E) A multiplicative-related bicluster. (F) A multiplicative-related bicluster in which the axis displayed in the PC plot is {C2/C1, C3/C1}.

In real situation, the arrangement of coherent genes and their corresponding conditions in biclusters is usually non-consecutive. Figure 3(A) shows an example where biclusters stay hidden and the special structures cannot be visualized easily. However, when the columns are rearranged as in Figure 3(B), we can see that the first five columns of the three rows (shown in red color) exhibit similar pattern. Therefore, columns re-arrangement is important in PC bicluster visualization. Once related columns in the dataset are found, the embedded special structures are revealed. This observation is utilized in the interactive adjustment of noise threshold for the biclustering algorithm proposed in the main article. Based on the columns in the identified biclusters, the expression values of related rows are examined to evaluate the homogeneity in the biclusters at a given noise threshold value.

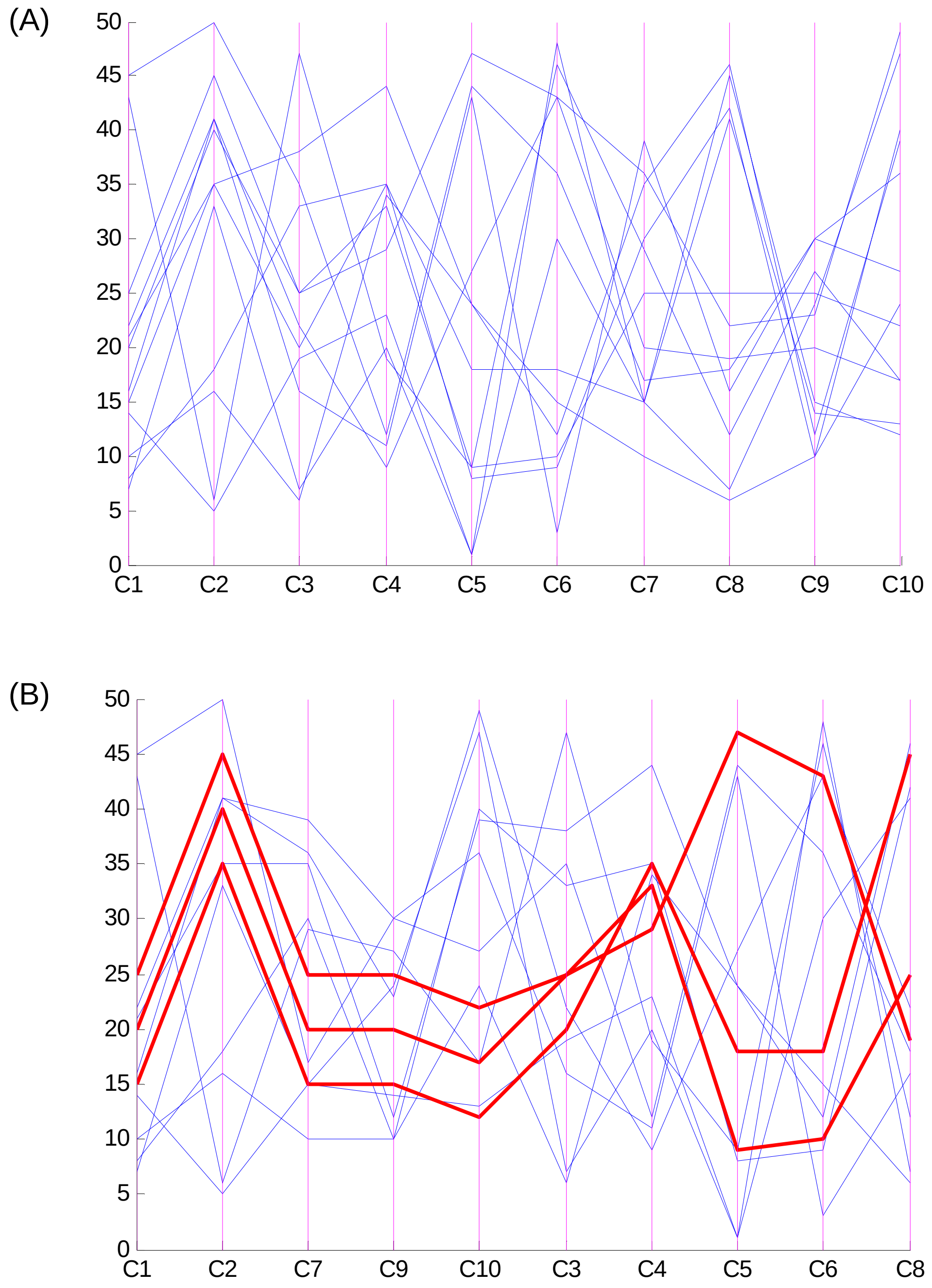

Figure 3 – Parallel coordinate plots for a 12 rows by 10 columns dataset. (A) Without column rearrangement. (B) With column rearrangement. After column rearrangement, the three rows (in red color in Figure (B)) that are related for columns {C1, C2, C7, C9, C10} can be seen easily.
